# Aligned fibrous scaffolds containing glycosaminoglycan mimetics enhance Schwann cell-supported axon growth and myelination

**DOI:** 10.1088/1741-2552/ae98b7

**Published:** 2026-09-03

**Authors:** Sharareh Hashemi, Sumayia Saif Jaima Chowdhury, Anna Kylat, Mary B Bunge, Martin Oudega, Treena L Arinzeh

**Affiliations:** 1 Biomedical Engineering, New Jersey Institute of Technology, Newark, NJ, United States of America; 2 Biomedical Engineering, Columbia University, New York, NY, United States of America; 3 The Miami Project to Cure Paralysis, University of Miami, Miami, FL, United States of America; 4 Physical Therapy & Human Movement Sciences, Northwestern University, Chicago, IL, United States of America; 5 Neuroscience, Northwestern University, Chicago, IL, United States of America; 6 Hines VA Hospital, Hines, IL, United States of America; 7 Shirley Ryan Ability Lab, Chicago, IL, United States of America

**Keywords:** astrocyte, axon growth, cellulose sulfate, dorsal root ganglion, glycosaminoglycans, neural tissue engineering, Schwann cell

## Abstract

**Objective.:**

Traumatic spinal cord injury causes functional impairments in large part due to the limited ability of damaged axons to regenerate. During development, the extracellular matrix (ECM) plays a critical role in providing molecular cues that promote and direct axon growth. Biomaterials that mimic the physicochemical properties of the ECM may be promising for promoting axon growth in the injured spinal cord. Glycosaminoglycans (GAGs) in the ECM during development are known to direct axon growth and guidance. We previously reported that GAG-mimetic cellulose sulfate, which can be synthesized with varying degrees of sulfation, partially and fully sulfated cellulose (pCelS and fCelS, respectively), promoted neurite extension over native GAGs.

**Approach.:**

The present study investigated the use of GAG-mimetic containing scaffolds as a platform for Schwann cell transplants to promote axon growth. The aligned fibrous scaffolds consisted of gelatin or gelatin blended with polycaprolactone (PCL), in order to improve hydrolytic stability, with 0.25 wt.% GAG-mimetics.

**Main results.:**

Our results demonstrated that the fCelS-containing scaffolds with SCs promoted more axon growth than the pCelS-containing scaffolds and the greatest myelination over all other scaffolds. Also, in the presence of astrocytes, fCelS-containing scaffolds supported axon growth. Furthermore, the fCelS-containing scaffolds were capable of binding and retaining a greater amount of the neurotrophins brain-derived neurotrophic factor and neurotrophin-3 as compared to all other scaffolds.

**Significance.:**

Aligned fibrous GAG-mimetic scaffolds containing fully sulfated cellulose may hold promise to repair damaged spinal cord tissue by providing physiochemical axon growth-promoting cues and a transplantation platform for SCs to support axon growth and myelination.

## 1. Introduction

Traumatic spinal cord injury (SCI) often results in axonal damage and consequently, loss of function [1–3]. Regenerating damaged axons may support recovery of function. During development, the extracellular matrix (ECM) provides key molecular cues that guide axon growth and thus, nervous tissue formation. Biomaterials approaches that mimic the physicochemical properties of the ECM during development may be a promising approach for promoting axon regeneration in the injured spinal cord [4–7]. Chondroitin sulfate proteoglycans (CSPGs) are known to either inhibit [8] or promote [9–11] axon growth. These different biological actions of CSPGs are determined by the sulfate patterns of the glycosaminoglycan (GAG) chondroitin sulfate (CS). In mammals, the sulfation pattern of CS changes from CS-C during early nervous tissue development to CS-A during the later developmental stages. CS-C facilitates axon growth [10], while CS-A inhibits axon growth and is present after injury in the glial scar [12, 13]. In previous studies, our group prepared electrospun aligned fibrous scaffolds, which have been shown to promote axon growth [14], composed of cellulose-derived glycosaminoglycan (GAG)-mimetics that consist of partially sulfated cellulose (pCelS), which has a sulfate predominately at the 6-carbon position of the glucose monomer unit, similar to CS-C, and fully sulfated cellulose sulfate (fCelS), which is sulfated at the 2-, 3-, and 6-carbon positions of the glucose monomer unit (supplement figure S1) [15]. The GAG-mimetic containing scaffolds improved axon growth over native CS-C and CS-A demonstrating the repair potential of the GAG-mimetics [15]. Findings demonstrated their promise as a scaffold for neural tissue engineering strategies.

In the present study, we investigated if aligned fibrous scaffolds containing GAG-mimetics, pCelS and fCelS, can be used as a platform for Schwann cell (SC) transplants. The goal of this study was to investigate if the scaffold-SC combination can promote axon growth. SCs support spinal cord repair by secreting axon growth-promoting molecules and myelinating axons [16]. SC transplantation in the injured spinal cord is accompanied often by improved functional recovery [17], and is feasible and safe for clinical application [18]. Our experiments were designed to test the hypothesis that aligned fibers containing cellulose derived GAG-mimetics and seeded with SCs can further increase axon growth and myelination. We also investigated SC-supported myelination of axons growing on the aligned fiber scaffolds and axon growth in the presence of unreactive astrocytes (Asts) as an explorative study to establish cytocompatibility of the scaffolds with glial cells of the CNS. The capacity of the GAG-mimetic containing fibers to bind brain-derived neurotrophic factor (BDNF) and neurotrophin-3 (NT-3) was also determined. BDNF and NT-3 are known axon growth-promoting neurotrophins and are secreted by SCs. The *in vitro* model consisted of dorsal root ganglion (DRG) neurons, which are routinely used for evaluating axon growth on biomaterials [14, 19, 20]. In addition, DRG neurons are pseudo-unipolar neurons and are used to study axon growth in both peripheral nervous system (PNS) [21, 22] and CNS [23, 24] applications, as they possess a peripheral axon and a central axon [25, 26]. Our findings supported our premise that aligned GAG-mimetic containing fibers can be used to deliver SCs and in the case of fCelS-containing scaffolds, promote the greatest SC-supported axon growth and myelination. These findings support that GAG-mimetic containing fiber scaffolds hold promise as a neural tissue engineering strategy to promote nervous tissue repair after SCI.

## 2. Material and methods

A full list of materials is provided in supplemental information (S.1).

### 2.1. Scaffold fabrication

24% (w/v) gelatin (Gel) and 22% (w/v) gelatin and polycaprolactone (Gel/PCL) polymer blend solutions with and without CelS were prepared. The Gel/PCL blend at the ratio of 80:20 was used to improve the handling and hydrolytic stability of Gel scaffolds (supplement figure S2 and table S1). Briefly, for Gel scaffolds, gelatin was dissolved in deionized water (DI) and acetic acid at 55 °C in water bath, followed by addition of 2,2,2-trifluoroethanol (TFE) at room temperature (RT) and stirred for 16–18 h. For Gel/PCL scaffolds, PCL was dissolved in TFE at RT before adding to the Gel solution. For scaffolds containing GAG-mimetics, 0.25% (w/w) pCelS or fCelS were first dissolved in DI water at 55 °C prior to addition of Gel or Gel/PCL.

The aligned fibrous scaffolds were electrospun in a closed chamber with humidity and ambient temperature of 33 ± 2% and 22 ± 2 °C, respectively. Briefly, the polymer solution was loaded into a syringe attached to a 16-G needle (Hamilton, NV, USA), with a positive voltage ranging from 12 to 23 kV and a flow rate of 0.5–0.8 ml h^−1^, using a syringe pump (New Era Pump Systems Inc. NY, USA). The aligned fibers were collected on a grounded mandrel rotating at 3200 rpm located at a distance of 20 cm from the needle tip. Scaffolds were then crosslinked using EDC and NHS in 100% ethanol for 96 h, based on the previously published protocol [27]. They were cut into 6- and 10-mm diameter disks using biopsy punches (Integra Miltex, York, PA), and dried using series of ethanol washes (30, 50, 70, and 100%). The optimal thickness of all hydrated scaffolds was approximately 0.3 ± 0.05 mm, measured with a caliper (VWR Traceable digital caliper, PA, USA). For *in vitro* cell culture studies, all scaffolds were sterilized with 100% ethanol three times for 10 min, rinsed three times with Dulbecco’s phosphate buffered saline (DPBS), transferred into the ultra-low attachment plates, and incubated in DPBS at 37 °C for 2 h. DPBS was replaced with the appropriate culture medium and incubated at 37 °C for 24 h prior to cell seeding. All experiments were performed three times for reproducibility. Gel and Gel/PCL scaffolds without the addition of the GAG-mimetic were used as negative controls in all experiments.

### 2.2. Fiber morphology and mechanical properties

All dried scaffolds were sputter coated (Bal-Tec 020 h, Leica Microsystems, Wetzlar, Germany) with gold/-palladium (6–8 *μ*m) at 40 uA for 20 s and imaged using scanning electron microscopy (SEM) (EM JSM-7900 F JEOL, USA) with accelerating voltage of 1–2 kV at average 10 mm working distance. A total of four images per scaffold were taken and the average fiber diameter (*n* = 40 fibers per scaffold) and degree of alignment (*n* = 20 fibers per scaffold) were measured using ImageJ software (1.52a, NIH, USA), as previously reported [28]. For mechanical testing, all crosslinked samples were hydrated in DPBS for 24 h and tensile testing was performed using Instron 3320 (Instron, MA, USA) at RT as previously reported [15]. A sample size of *n* = 7 per group was used to measure Young’s modulus and ultimate tensile stress.

### 2.3. Fourier transform infrared spectroscopy (FTIR)

The presence of GAG-mimetics and PCL in the Gel fibers was detected using FTIR spectroscopy (Perkin-Elmer FTIR-ATR 100 series, Perkin Elmer Life and Analytical science, Shelton, CT, USA). Data were collected in the 500–1800 cm^*−*1^ range with a resolution of 4 cm^*−*1^ and total scan of 40. FTIR was also performed for PCL scaffolds alone.

### 2.4. Swelling behavior and hydrolytic stability

The swelling behavior and hydrolytic stability of all crosslinked scaffolds were investigated for up to 8 weeks (*n* = 4 per group per time point). The swelling behavior was evaluated as previously published protocol [29]. Briefly, dried scaffolds were weighed (*w*_initial_) and then scaffolds were weighed after being hydrated in DPBS at 37 °C (*w*_Hydrated_) at 10 min, 1 h, 4 h, and 1, 4, 7, 14, 21, 28, 35, 42, 49, and 56 d. Swelling was calculated as % Swelling = [(*W*_Hydrated_—*W*_Initial_)/*W*_Initial_] * 100. After 8 weeks, all scaffolds were dried using series of ethanol washes and changes in fiber morphology were evaluated using SEM as described above.

For hydrolytic stability, all dried scaffolds were first weighed and then immersed in Minimum Essential Media (MEM) containing 10% heat inactivated fetal bovine serum (HI-FBS) and 1% penicillin-streptomycin and incubated at 37 °C. The media was collected, and fresh media was added at the selected time intervals, days 1, 4, 7, 14, 21, 28, 35, 42, 49, and 56. The amount of gelatin dissolved in the media at each time point was quantified using Micro BCA protein assay kit according to the manufacturer’s protocols using gelatin as a standard. The absorbance was measured at 570 nm using a microplate reader, Emax (Molecular Devices, San Jose, CA, USA). A standard curve was used to determine the gelatin amount. The percent loss was calculated based on the amount of dissolved gelatin in the media normalized to the original weight (dried) of the gelatin in the scaffolds.

### 2.5. Neurotrophin binding and release

Our previous study showed that nerve growth factor interacts with GAG-mimetic scaffolds with a higher amount binding to scaffolds containing fCelS in comparison to scaffolds containing pCelS or native GAGs [15]. SCs are known to secrete a variety of neurotrophins [15] that could stimulate axon growth. Therefore, all scaffolds were evaluated for the binding and release of BDNF and NT-3. Dried scaffolds (*n* = 5 per group), 6 mm diameter disks, were hydrated in DPBS at 37 °C for 24 h, then incubated in 100 ng ml^*−*1^ of either human NT-3 or human BDNF prepared in 0.1% bovine serum albumin (BSA) (approximately 10 ng protein/scaffold) at 37 °C for 24 h. The amount of bound neurotrophin was determined following previously published protocols using the pull-down assay [30]. Briefly, the total amount of neurotrophins in the supernatant was measured using enzyme-linked immunosorbent assay (ELISA) kits for NT-3 and BDNF protein following the manufacturer’s protocols. Absorbance was measured at 450 nm using FLX800 microplate reader (Biotek Instruments, VT, USA) at 480 nm excitation and 520 nm emission. The amount of bound protein to the scaffold was determined by subtracting the amount in the supernatant at 24 h from the initial concentration. For the release of neurotrophins, following the incubation of scaffolds with neurotrophins for 24 h as described, all scaffolds were then incubated in MEM containing 10% HI-FBS and 1% penicillin-streptomycin at 37 °C for one week to determine the release of the neurotrophins in the media at days 1, 4 and 7 and quantified using the ELISA kits as described above. The percentage of cumulative release of neurotrophins at each time point was calculated as sum of the total released over time divided by initial amount adsorbed by each scaffold.

### 2.6. SC and astrocyte (Ast) growth on scaffolds

The methods for the SC and Ast isolation and culture media can be found in supplemental information (S.2). The effect of scaffolds on SC and Ast growth was evaluated for up to 14 and 4 d, respectively. The 6 mm scaffolds were pre-treated in SC media or Ast media. eGFP-SCs at passage 3–4 or Asts at passage 1 were seeded at the density of 71.4 K cells cm^*−*2^ and 35.7 K cells cm^*−*2^, respectively, per scaffold. Media was changed every 2–3 d and cell number was determined at each time point using Quant-iT dsDNA Picogreen Assay kit using a known cell number for standards. All samples and standards were lysed in Papain protease solution and fluorescence intensity was measured as previously described [29]. At each time point, *n* = 4 samples per group were used for cell number and *n* = 2 samples per group were fixed and imaged using confocal microscope (EZ-C1 Nikon, USA). A description of the immunostaining methods is provided in supplemental information (S.3).

### 2.7. DRG axon growth on scaffolds

DRG isolation and media are described in supplemental information (S.2). DRG explants were seeded directly on 6 mm disk scaffolds and allowed to attach for 3 h in NB medium. Then media was replaced with NBF media to minimize endogenous SCs and fibroblasts in the culture. After 3 d, all samples were fixed, immunostained, and imaged as previously described [15]. The lengths of 10 axons per scaffold were measured, at random, on each side of the DRG body, from perimeter of the DRG body to the tip using freehand line tool in ImageJ and then averaged per scaffold. *n* = 4 per group were used.

### 2.8. DRG co-culture with SCs or Asts

To more closely mimic axon growth after injury, DRGs were purified prior to seeding on scaffolds as previously reported [31]. Briefly, DRGs were seeded on collagen coated plates after isolation, and the purification procedure was performed by alternating between NB and NBF media for one week, and culture was maintained in NB media for one more week. eGFP-SCs or Asts were seeded at density of 221.5 K cells cm^*−*2^ on 10 mm disk scaffolds (*n* = 4 scaffolds per group). After 24 h, purified DRGs were axotomized and seeded on eGFP-SC- or Ast-laden scaffolds. The co-culture was maintained in CN media. Axotomized DRGs seeded onto scaffolds, without eGFP-SCs or Asts (*n* = 4 scaffolds per group), were maintained in NB media. Cultures were maintained for 3 d, fixed, immunostained for neurofilament (NF) and GFAP and imaged using confocal microscopy. Length of axons was measured as described above using the same sample size, unless otherwise noted.

### 2.9. Myelination study

This study was performed on scaffolds, 10 mm diameter disks, and 12 mm coverslips as control. All scaffolds were pre-treated in DN media. The 12 mm coverslips were pre-coated overnight with matrigel (250 *μ*g ml^*−*1^ in NB medium). The DRG neuron purification and myelination was established as reported elsewhere [32]. Briefly, isolated DRGs were dissociated and seeded as 4 or 1.5 DRGs per scaffold or per coverslip, respectively. Following the purification process, by day 16, the exogenous SCs at density of 221.5 K cells cm^*−*2^ were added to the purified neurons and the culture was maintained in CM media for up to 23 d. Then media was replaced with CM media containing 50 *μ*g ml^*−*1^ of ascorbic acid (CM/AA media), and the culture was maintained for 2–3 weeks. Cultures were fixed and immunostained for NF and myelin basic protein (MBP). The length of myelinated segments on each scaffolds was measured using ImageJ. *N* = 4 samples per group and *n* = 10 myelinated segments per sample were measured and averaged per sample. *N* = 4 samples per group were used to quantify the amount of MBP using MBP-ELISA kit. Briefly, the samples were lysed using Cell Extraction Buffer following the manufacturer protocol. The total amount of extracted protein was quantified using Micro BCA protein assay kit following the manufacturer’s protocol and equal amounts of protein from each group was used for the MBP-ELISA kit following the manufacturer protocol. The amount of Glyceraldehyde 3-phosphate dehydrogenase (GAPDH) was measured as an internal control. The ratio of MBP:GAPDH was calculated for all samples.

### 2.10. Statistical analysis

Statistical analysis was performed using one-way and two-way analysis of variance (ANOVA) followed by the post hoc Tukey’s HSD planned comparison test (using Graphpad Prism software) to determine statistical differences between groups and time at *p* < 0.05.

## 3. Results

### 3.1. Scaffold morphology and mechanical properties

Aligned fibrous scaffolds were successfully fabricated. Gelatin scaffolds with or without partially or fully sulfated cellulose are denoted as Gel/pCelS, Gel/fCelS, and Gel. Gel/PCL blend scaffolds with or without partially or fully sulfated cellulose are denoted as Gel/PCL/pCelS, Gel/PCL/fCelS, and Gel/PCL. The SEM images of all scaffolds before and after crosslinking are shown in figure 1. All scaffolds showed unidirectional orientation. GAG-mimetic scaffolds had larger fiber diameters after crosslinking (table 1).

No significant differences among groups were detected for fiber diameters before crosslinking. However, after crosslinking the fiber diameter of scaffolds containing CelS was significantly higher than Gel and Gel/PCL alone. Also, all Gel/PCL scaffolds with or without CelS had higher tensile properties in comparison to all Gel scaffolds with or without CelS.

### 3.2. FTIR spectroscopy

Sulfate peaks due to S=O stretch of sulfate ester in CelS occur in the range of 1200–1235 cm^*−*1^ [33]. A small peak at about 1200 cm^*−*1^ for both Gel/pCelS and Gel/fCelS was detected, and it was slightly more pronounced for fCelS (supplement figures S3(A) and (B)). A similar peak was detected at about 1190 cm^*−*1^ in Gel/PCL scaffolds containing CelS, which corresponds to S=O stretch and may be shifted due to the presence of the sharper peak for PCL at about 1165 cm^*−*1^.

### 3.3. Addition of the GAG-mimetic increased the swelling behavior of scaffolds

A significant increase in swelling (220 ± 20%) was observed within the first 10 min of incubation for all scaffolds (supplement figure S4). The % swelling for all other time points (up to 8 weeks or 1344 h) was normalized to the % swelling at 10 min of immersion. By day 21 (504 h), Gel scaffolds containing CelS had significant swelling where Gel/fCelS was significantly higher than all other groups (supplement figure S4(A)). The significant swelling in Gel scaffolds containing CelS made handling difficult, therefore, the percentage of swelling for these samples was reported for up to 21 d only. No significant changes in swelling were observed after day 28 (672 h) for all other scaffolds over time, but the overall percentage of swelling for Gel/PCL scaffolds containing CelS stayed significantly higher in comparison to Gel and Gel/PCL at each time point after day 28. As shown in supplement figure S4(B), fiber morphology was retained for all scaffolds after 8 weeks, but to a lesser extent for Gel/fCelS group.

### 3.4. Addition of PCL improved hydrolytic stability of scaffolds

The overall protein loss for all Gel scaffolds was significantly higher than their corresponding Gel/PCL scaffolds at each time point (supplement figure S5). The protein loss for all Gel scaffolds was around 0.7 ± 0.05% at day 1 with a cumulative loss of 5 ± 0.4% by day 56, while all Gel/PCL scaffolds lost 0.35 ± 0.03% at day 1 with a cumulative loss of 3 ± 0.5% by day 56. The protein loss for Gel/PCL/fCelS scaffolds was significantly lower than all other groups until day 28. The cumulative protein loss for Gel/PCL/fCelS scaffolds was 1.4% by day 28 and it gradually increased to 2% by day 56.

### 3.5. Addition of the GAG-mimetic fCelS improved BDNF and NT-3 binding and release

All scaffolds with or without CelS were able to sequester BDNF and NT-3 (supplement figures S6(A) and (B)). The amount of NT-3 bound to the scaffolds containing fCelS was significantly higher compared to all other groups. For BDNF sequestration, the gelatin scaffolds containing fCelS was significantly higher than all other scaffolds. All scaffolds were able to sequester approximately 8–9 ng of NT-3 and 7 ng of BDNF (out of 10 ng per scaffold added originally). The cumulative release profile for BDNF and NT-3 is shown in supplement figures S6(C) and (D). At day 1, scaffolds containing fCelS released approximately 30% and increased up to 50% by day 7. Whereas, for all other scaffolds, approximately 80% of BDNF and NT-3 was released by day 7.

### 3.6. fCelS-containing scaffolds enhanced axon growth

DRG explants attached to all scaffolds (supplement figures S7(A) and (B)). The axon length was significantly higher on all Gel scaffolds in comparison to all Gel/PCL scaffolds with or without CelS (supplement figure S7(C)). Axons were extended along the length of fibers for all groups. Gel/fCelS scaffold groups had significantly longer axon length than all other groups. All scaffolds supported axotomized DRG attachment and axon outgrowth (figure 2(A)). A representative image of Gel/fCelS scaffold groups at higher magnification is shown in figure 2(B). Axon length was significantly longer on Gel/fCelS scaffolds (figure 2(C)) in comparison to all other groups. Axon length on Gel/PCL/fCelS scaffolds was significantly longer than Gel and Gel/PCL groups (figure 2(C)).

### 3.7. All scaffolds supported SC growth and SC-mediated axon growth with the greatest for fCelS-containing scaffolds over gelatin alone and pCelS-containing scaffolds

All scaffolds supported SC attachment and growth. SCs morphology at 3 and 24 h is shown in figure 3. SCs appeared to have spindle-shape cell body with extended bipolar processes along the length of fibers (figure 3(A)). Fiber alignment induced bipolar cell morphology as early as 3 h. SC process length at 24 h was significantly longer on Gel/fCelS group as compared to the Gel/PCL/fCelS group (figure 3(B)).

A representative image of SCs on Gel/fCelS scaffolds at a higher magnification at day 14 is shown in figure 3(C). Scaffold fibers are autofluorescent in blue however fibers are not distinguishable possibly due to swelling. Images of SCs on all scaffolds at each time point are found in supplement figure S8. Significantly higher cell number was detected on Gel/fCelS scaffolds in comparison to Gel, Gel/PCL and Gel/PCL/fCelS scaffolds at day 4 and cell number did not increase for the Gel/fCelS group from days 4 to 14 (figure 3(D)). SCs numbers increased significantly for all other groups between days 4 and 14.

In co-cultures containing SCs and axotomized DRGs, axons extended on all scaffolds at 3 d (figure 4(A)). SCs, with elongated processes, were in close contact along the length of axons and scaffold fibers (figure 4(B)). Axon length was significantly higher on scaffolds containing fCelS in comparison to those containing pCelS (figure 4(C)). For all groups, the length of axons was three times longer than those cultured on scaffolds in the absence of SCs (figures 4(C) and 2(C)).

### 3.8. fCelS-containing scaffolds supported Ast-mediated axon growth but without ast growth

Since fCelS-containing scaffolds promoted axon growth in SC/DRG co-culture, they were investigated for Ast attachment and Ast-supported axon growth. Gel scaffolds were also used as a control. All three scaffolds supported Ast attachment (figure 5(A)). Asts at day 1 had elongated morphology along the length of the fiber. The elongated morphology was more pronounced on both scaffolds containing fCelS in comparison to Gel scaffolds. GFAP expression appeared to be more pronounced at day 1 and declined by day 4 on all scaffolds. Asts showed a spread morphology on all scaffolds at day 4. The cell number increased on Gel scaffolds by day 4, but it did not significantly change on both fCelS-containing scaffolds (figure 5(B)).

Representative images of fCelS-containing scaffolds are shown in figure 5(C). All scaffolds supported axon growth in the presence of Asts by day 3. Axons were elongated along the length of aligned fibers for all scaffolds with the shortest length on Gel/PCL/fCelS scaffolds (figures 5(C) and (E)), No significant differences were detected between Gel and Gel/fCelS scaffolds. Asts were seen interacting with axons for all scaffolds (figure 5(D)).

### 3.9. fCelS-containing scaffolds enhanced SC myelination

Myelination occurred as indicated by MBP staining for all groups at 37 d of culture (figure 6(A)). Myelinating segments were generally parallel to each other and in the direction of fiber alignment (figure 6(B)). Length of myelinated segments was longer on Gel/fCelS scaffolds than pCelS containing scaffolds at 37 d (figure 6(C)).

Caspr, a marker of paranodal domains representing the node of Ranvier, was detected on all scaffolds at 44 d of culture, where figure 6(D). Shows a representative image of Caspr/MBP staining for Gel/fCelS scaffolds. The amount of MBP expression normalized to GAPDH after 44 d culture is shown in figure 6(E). The MBP level indicating the degree of myelination was significantly higher on fCelS scaffolds in comparison to all other groups.

## 4. Discussion

The limited regenerative capacity of the spinal cord after injury creates a significant challenge for successful axonal regeneration. In several studies, it was shown that SC transplants in the damaged spinal cord support axon growth; however, the overall extent of growth remains limited. Here, we have built on the key role of ECM-associated GAGs in axon growth in the developing central nervous tissue [34–36] and fabricated aligned fibrous scaffolds containing GAG-mimetics that have axon growth-promoting properties. Our data demonstrated the efficacy of SC-seeded aligned fibrous GAG-mimetic scaffolds where fCelS was more favorable for both SC-supported axon growth and myelination. Similarly, axon growth was also shown on aligned fibrous GAG-mimetic scaffolds seeded with Asts. These findings support the potential of this neural engineering approach for eliciting repair in the injured spinal cord.

The scaffolds containing the sulfated GAG-mimetics supported neurotrophin binding. Neurotrophins, such as BDNF and NT-3, bind to the native CS, with greater affinity for highly sulfated CS-E [37]. Here, we found that fibrous scaffolds containing the highly sulfated fCelS had greater BDNF and NT-3 binding and retained both neurotrophins better than scaffolds with pCelS. Heparin is the highest sulfated GAG known in nature and it has been studied for its ability to foster axon growth in the damaged nervous tissue because of its high affinity to bind neurotrophins and other types of growth factors [37, 38]. In our study, we also found that axon growth was highest on SC-seeded fCelS containing scaffolds as compared to gelatin alone and all pCelS-containing scaffolds. Possibly, the scaffolds bound and retained BDNF and NT-3 secreted by the SCs [39, 40].

The differences in SC, Asts, and axon growth on Gel vs. Gel/PCL with or without CelS may be due to differences in surface chemistry, which has been shown to influence cell attachment and function [41] and Ast reactivity [42–44]. The addition of fCelS to Gel or Gel/PCL blend scaffolds improved axon growth with or without SCs, although not significantly greater for the SC-seeded Gel/PCL/fCelS scaffolds in comparison to Gel/PCL. In addition, scaffolds containing fCelS did not enhance Ast growth over the time points observed. The fCelS, which is a fully sulfated GAG-mimetic with a higher degree of sulfation than native GAGs, may be more favorable at supporting axon growth and in the presence of Asts, in contrast to the sulfated GAGs in the glial scar. An increase in Ast cell number and GFAP expression correlate with reactive astrogliosis, glial scarring [45]. Following SCI, Asts express a wide range of ECM molecules including CSPGs, contributing to the formation of the glial scar impeding axon growth [46]. In this study, the length of axons grown on scaffolds containing fCelS in the presence of Asts was at least 1.5 mm shorter than those grown in the presence of SCs; however, it was still about 1 mm longer than axons grown in culture alone. Both SCs and Asts can produce neurotrophins that are favorable for axon growth [46]. These results indicate that the Asts grown on these fibrous scaffolds provide a supportive environment for axon growth. Further investigation is needed to evaluate the effect of these scaffolds in the presence of reactive Asts, using stimulators such as pro-inflammatory cytokines, and in co-cultures that include both SCs and reactive Asts. Physical cues from the fibers may have also had an effect on SC and Ast behavior as well as axon growth where a significant increase in fiber diameters were determined for CelS-containing scaffolds and increased mechanical properties with the addition of PCL. Previous studies have demonstrated that fiber diameters and mechanical properties can influence glial cell behavior [47] and axon growth [14].

The co-culture of DRGs and SCs have been widely used as an *in vitro* model to study myelination [20, 48]. In this study, the GAG-mimetic containing scaffolds using dissociated DRGs co-cultured with primary adult SCs supported SC-mediated myelination. All scaffolds in this study were able to provide adequate SC adhesion to support myelination, where those containing fCelS showed higher expression of MBP, the major protein component in both PNS and CNS [49, 50], indicating an enhancement in SC myelination. The fCelS containing scaffolds enhanced neurotrophin binding and retention, which may also have an effect on promoting myelination. Similarly, SC processes were significantly longer on Gel/fCelS scaffolds. Elongation of SC processes is important to initiate myelination [51] and transplanted SCs have been shown to remyelinate host axons following SCI [52].

Sulfated GAGs are present in the CNS, and their expression varies during development and in response to injury [10, 12]. The effect of sulfated GAGs on cell function depends on their length, sulfate position and the degree of sulfation governing their binding affinity to soluble bioactive molecules and cell surface receptors to either promote or inhibit axon growth [11, 37, 53, 54]. In our previous study [15], scaffolds containing CelS with 0.76 and 2.3 sulfates per monosaccharide had higher binding of NGF over scaffolds containing native GAGs, including CS-A and CS-C, which have 0.32 and 0.54 sulfates per monosaccharide, respectively. Axon growth also increased as the degree of sulfation increased; however, no significant differences in axon growth were detected between the GAG-mimetics. In this study, scaffolds containing fCelS retained neurotrophins and promoted axon growth, SC-mediated axon growth and myelination over the pCelS-containing scaffolds. Scaffolds containing pCelS, with 0.54 sulfate group per monosaccharide unit similar to native CS-C, with or without SCs did not show significant differences or an increase in axon growth in comparison to Gel or Gel/PCL scaffolds. These results indicate the effect of scaffolds containing pCelS on axon growth and myelination may depend on SC function. It should be noted the amount of CelS used in this study was based on the amount of sulfated GAGs present in decellularized neural tissue in the rat [55], where this amount may not be enough for scaffolds containing pCelS to have an effect on axon growth with or without SCs in comparison to Gel or Gel/PCL scaffolds. Future studies may need to examine a greater amount of pCelS in the scaffolds.

The major goal of this study was to design and fabricate a biodegradable fibrous construct that could promote SC-mediated axon growth and myelination. Our findings demonstrated that the fibrous, GAG-mimetic containing scaffold, specifically the fully sulfated GAG-mimetic, may hold promise for enhancing growth of damaged axons with or without SCs and enhance axon myelination by seeded SCs. The effects that were observed depended on the degree of sulfation of the GAGs. The addition of PCL to the scaffold improved mechanical properties and hydrolytic stability in comparison to Gel alone and SCs were needed to improve axon growth, which may have implications when using *in vivo*. In future work, scaffolds containing GAG-mimetics may need to be further optimized for investigation in *in vivo* SCI models.

## 5. Conclusion

This study demonstrated fibrous scaffolds containing GAG-mimetics enhanced axon growth with or without SCs and SC-mediated myelination, indicating the scaffolds may have potential use in combination with SCs as a therapeutic strategy for spinal cord repair. Scaffolds containing fully sulfated cellulose were most favorable in supporting axon growth with or without SCs, which may be a result of enhanced binding and retention of neurotrophins. In addition, scaffolds when in combination with Asts supported axon growth, which suggests they may provide a permissive environment for axon growth *in vivo*. This study demonstrates the potential of GAG-mimetic containing scaffolds in combination with SCs for spinal cord repair.

## Supplementary Material

Supplemental Material

Supplementary material for this article is available online

## Figures and Tables

**Figure 1.**
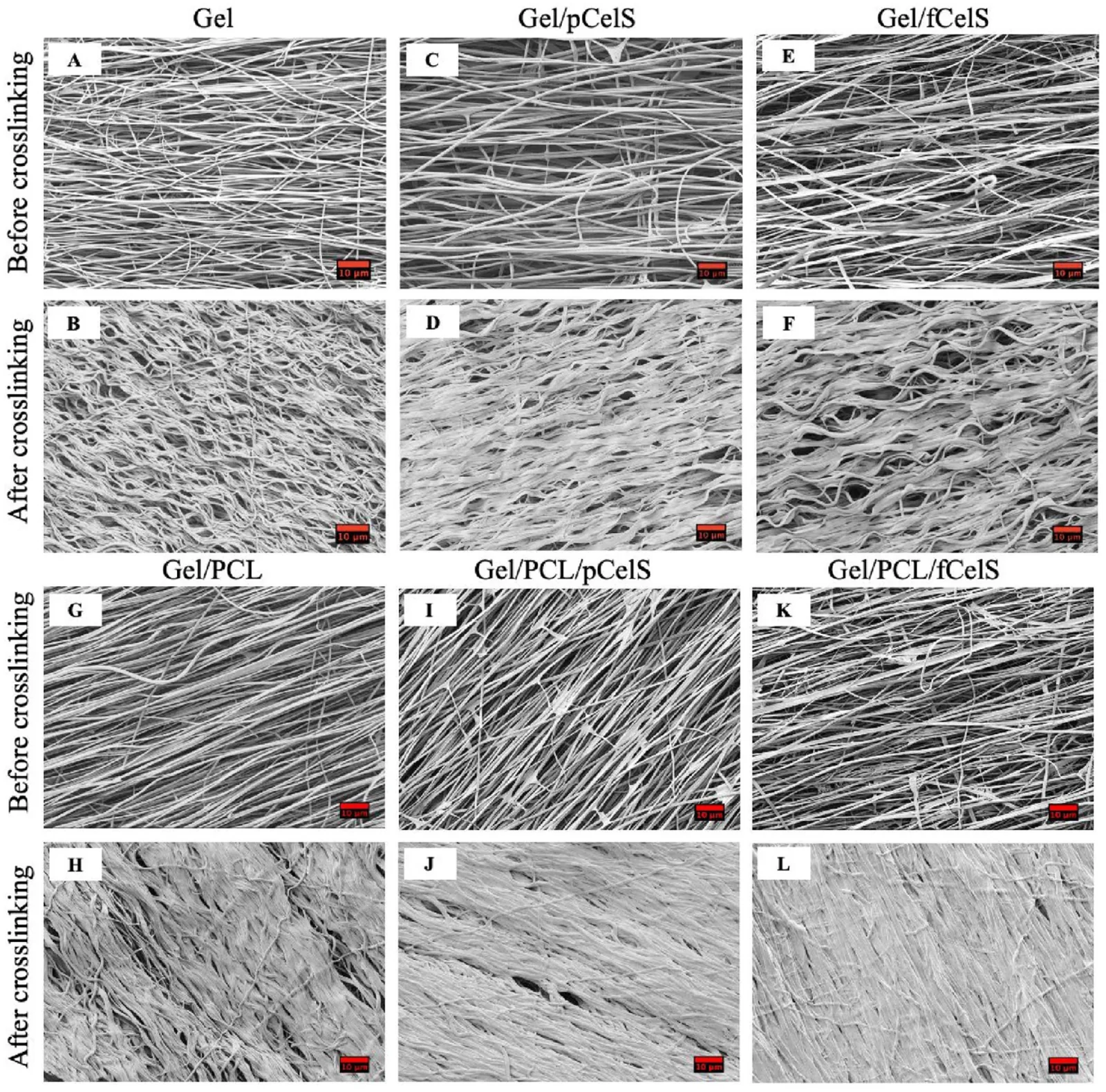
Representative SEM images of all scaffolds before and after crosslinking, respectively. (A) and (B) gelatin (Gel), (C) and (D) gelatin with partially sulfated cellulose (Gel/pCelS), (E) and (F) gelatin with fully sulfated cellulose (Gel/fCelS), (G) and (H) gelatin with polycaprolactone (Gel/PCL), (I) and (J) gelatin with polycaprolactone and partially sulfated cellulose (Gel/PCL/pCelS), and (K) and (L) gelatin with polycaprolactone and partially sulfated cellulose (Gel/PCL/fCels); Mag.: 2000X; Scale bar: 10 *μ*m.

**Figure 2.**
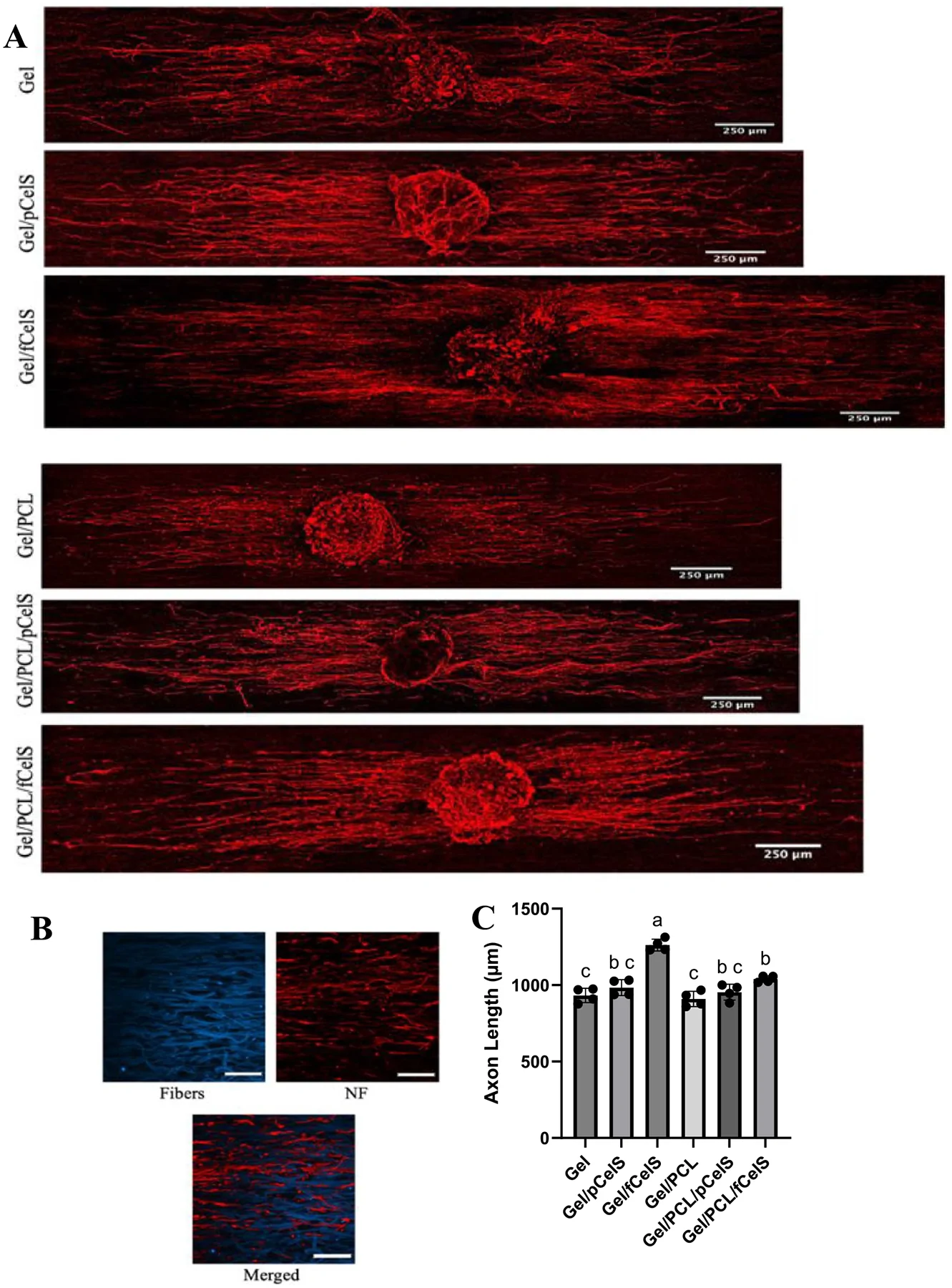
(A) Representative merged confocal images of axotomized DRGs alone on all scaffolds at 3 d in culture; Scale bar: 250 *μ*m; Red = Neurofilament. (B) A representative confocal image on Gel/fCelS scaffolds at Mag. 60X; Scale bar: 50 *μ*m; Fibers are autofluorescent in blue; Red = Neurofilament. (C) Axon length on all scaffolds; *n* = 4 per group where each data point represents the average of 10 axons per sample; Groups with different letters are statistically significant (*p* < 0.05). Column scatter plot where the bar and error bars represent the Mean ± S.D.

**Figure 3.**
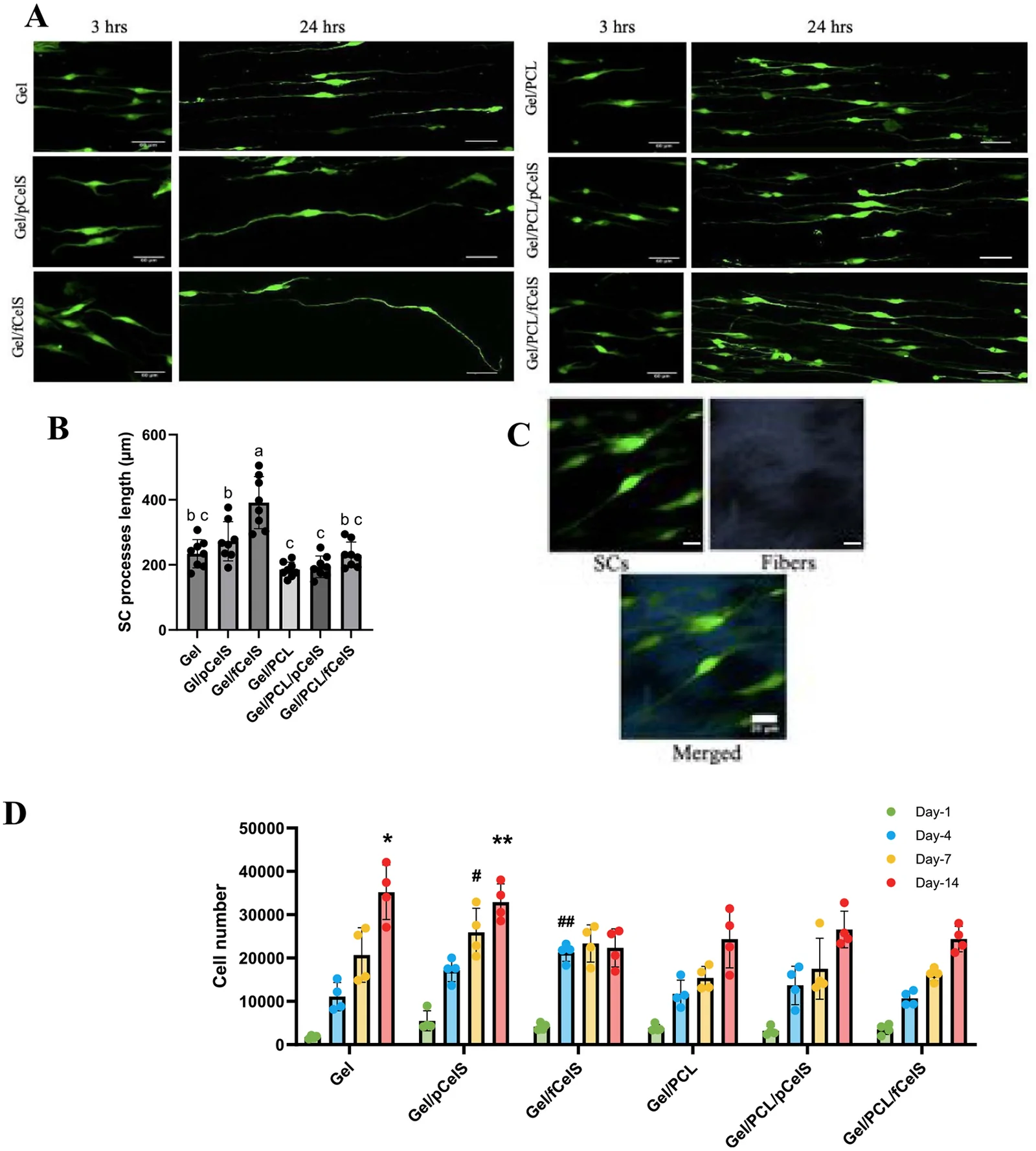
(A) Representative confocal images of eGFP-SCs on all scaffolds after 3 and 24 h; Scale bar: 60 *μ*m. (B) Length of processes after 24 h; Groups with different letters are statistically significant (*p* < 0.05); *n* = 8 per group. (C) A representative confocal image of eGFP-SC on Gel/fCelS scaffolds at day 14; Mag. 100X; Scale bar: 20 *μ*m; Fibers are autofluorescent in blue. (D) SC cell number at each time point; *n* = 4 scaffolds per group; ## Significantly higher cell number for Gel/fCelS scaffolds in comparison to Gel, Gel/PCL and Gel/PCL/fCelS scaffolds at day 4 (*p* < 0.05); # Significantly higher cell number for Gel/pCelS in comparison to all Gel/PCL groups at day 7 (*p* < 0.05); *Significantly higher cell number for Gel scaffolds in comparison to all other scaffolds except for Gel/pCelS at day 14 (*p* < 0.05); **Significantly higher cell number for Gel/pCelS in comparison to Gel/fCelS, Gel/PCL and Gel/PCL/fCelS at day 14 (*p* < 0.05). All groups increased in cell number from days 4 to 14, except for Gel/fCelS group (*p* < 0.05). All graphs are column scatter plots where the bars and error bars represent the Mean ± S.D.

**Figure 4.**
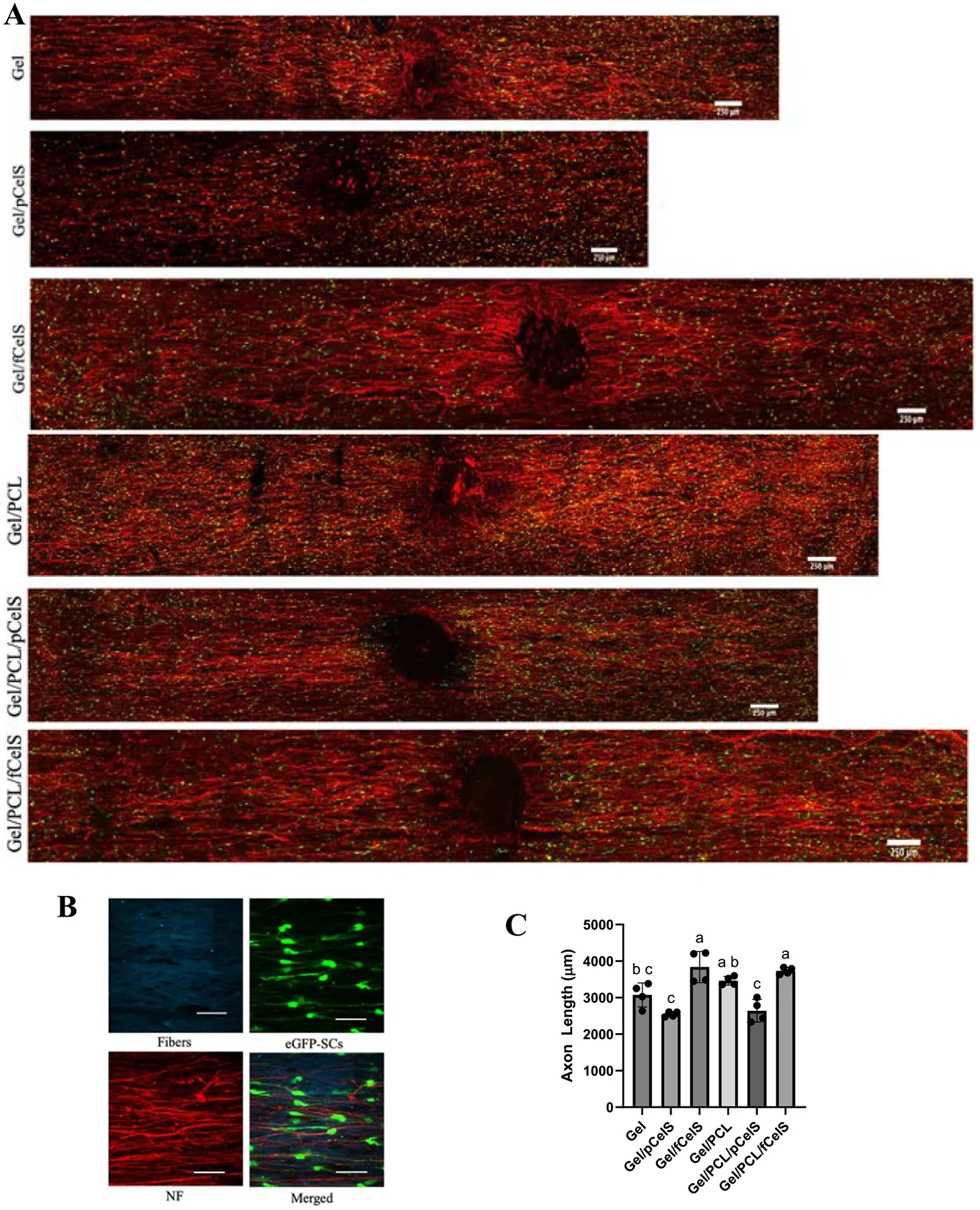
(A) Representative merged confocal images of purified and axotomized DRGs co-cultured with exogenous eGFP-SCs on all scaffolds at day 3; Red: Neurofilament; Green: SCs; Fibers are autofluorescent in blue; Scale bar: 250 *μ*m. (B) A representative confocal image on Gel/fCelS scaffolds at Mag. 60X; Scale bar: 50 *μ*m; Fibers are autofluorescent in blue. (C) Axon length on all scaffolds; *n* = 4 per group where each data point represents the average of 10 axons per sample; Groups with different letters are statistically significant (*p* < 0.05). Column scatter plot where the bar and error bars represent the Mean + S.D.

**Figure 5.**
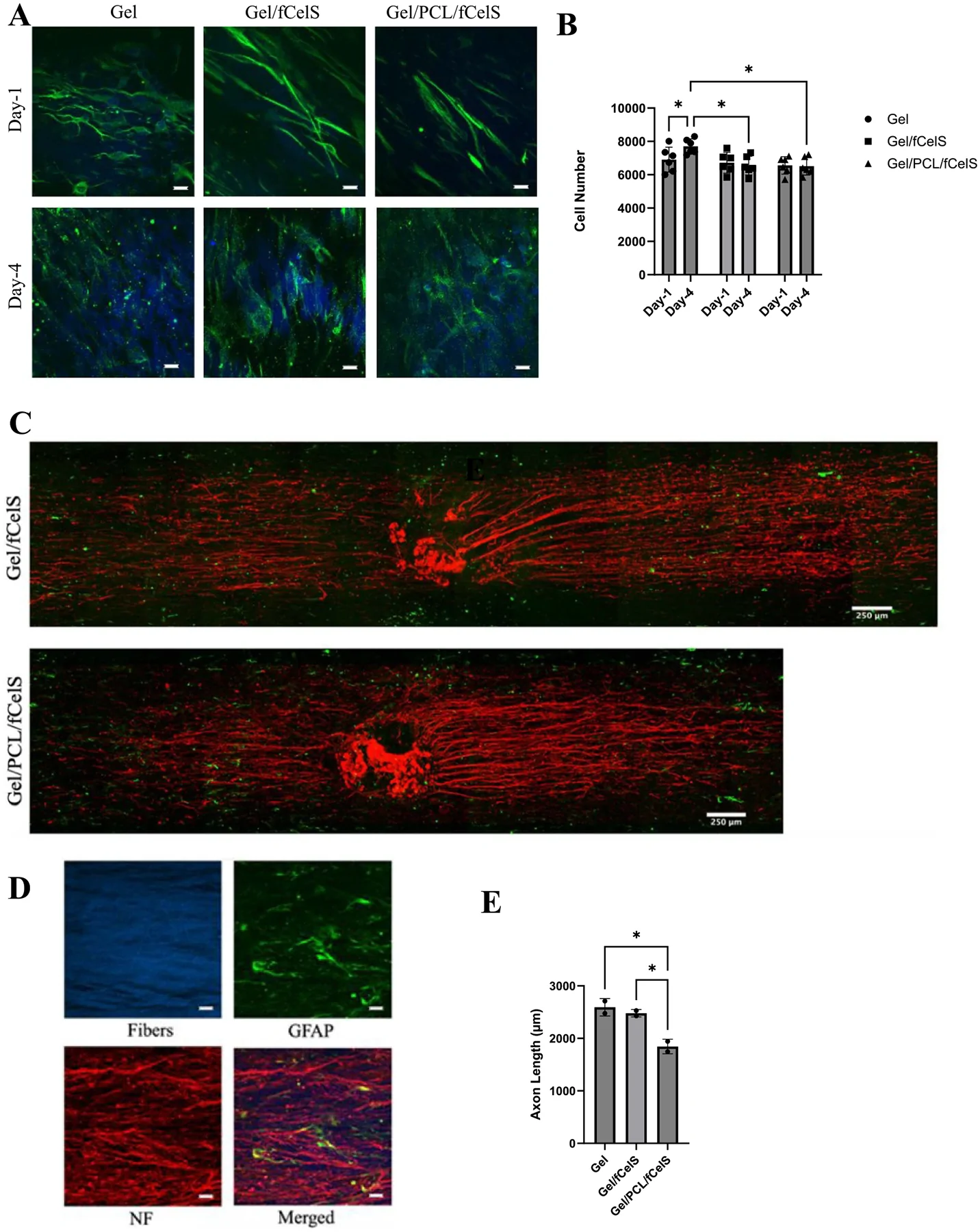
(A) Representative merged confocal images of astrocytes (Asts) at each time point; Mag. 60X; Scale bar: 50 *μ*m; Green: GFAP; Blue: Dapi, Fibers are autofluorescence in blue. (B) Ast cell number at each time point; * *p* < 0.05; *n* = 6 per group. (C) Representative merged confocal images of purified axotomized DRGs co-cultured with Asts at day 3; Red: Neurofilament; Green: GFAP; Scale bar: 250 *μ*m. (D) A representative confocal image on Gel/fCelS scaffolds at higher magnification, Mag. 60X; Scale bar: 50 *μ*m; Fibers are autofluorescent in blue. (E) Axon length on all scaffolds at day 3; *n* = 2 per group where each data point represents the average of 10 axons per sample; *p < 0.05. Column scatter plot where the bar and error bars represent the Mean ± S.D.

**Figure 6.**
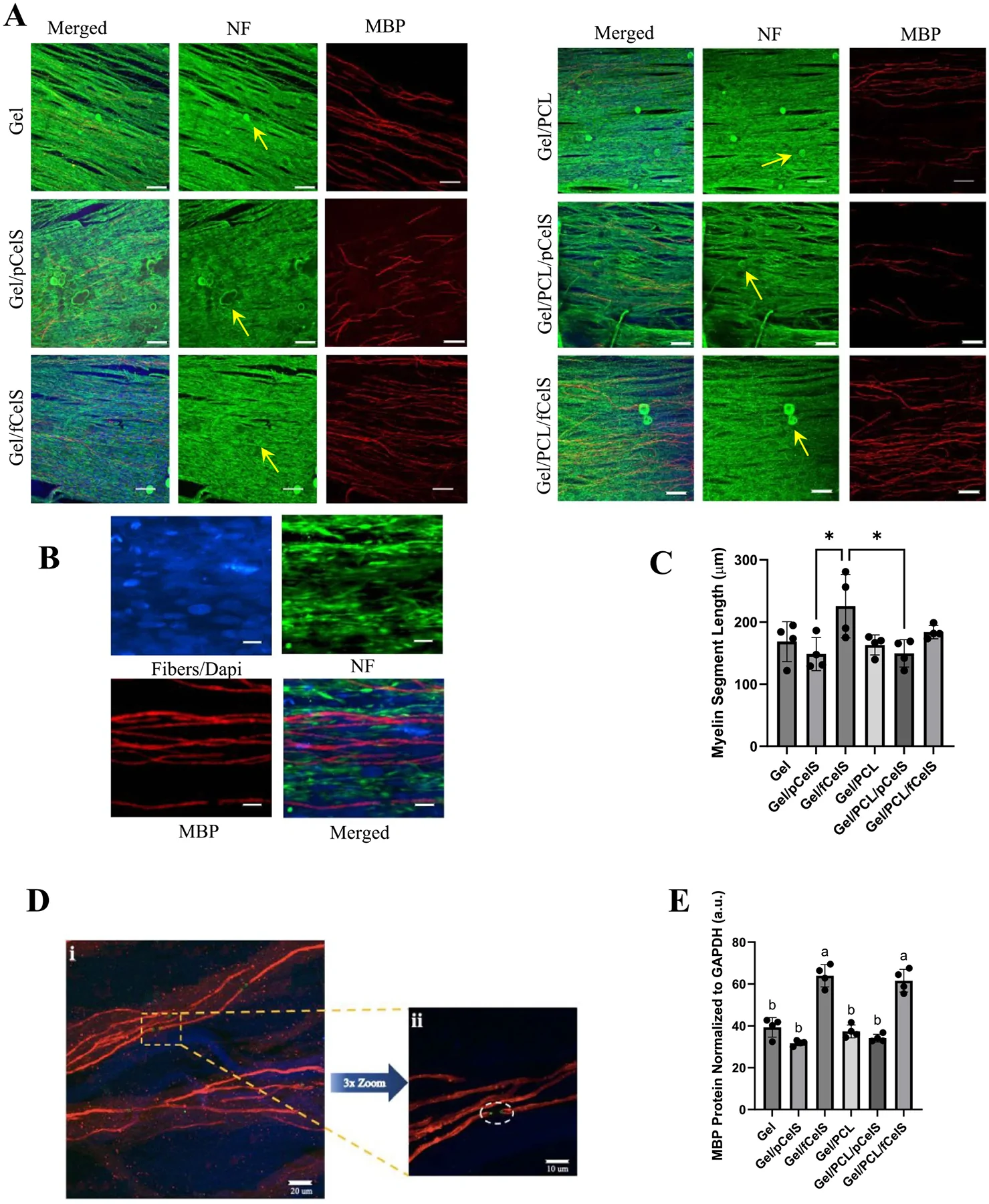
(A) Representative confocal images of SC-myelination on all groups; Mag. 20X; Scale bar: 100 *μ*m. at day 37. Yellow arrows show neuron cell bodies. (B) A representative confocal image of Gel/fCelS scaffolds at Mag. 100X (4x zoom); Scale bar: 5 *μ*m. Green: Neurofilament; Red: MBP; Blue: Dapi, Fibers are autofluorescent in blue. (C) Length of myelin segments on all groups at day 37; *n* = 4 per group where each data point represents the average of 10 segments per sample *p < 0.05. (D) A representative confocal image of Caspr on Gel/fCelS scaffolds at day 44; (i) Mag. 60X; Scale bar: 20 *μ*m; (ii) 3x zoom; Scale bar: 10 *μ*m; Green: Caspr, Red: MBP. (E) The amount of MBP protein normalized to GAPDH at day 37; *n* = 4 per group; Groups with different letters are statistically significant (*p* < 0.05). Column scatter plot where the bar and error bars represent the Mean ± S.D.

**Table 1.** Scaffold morphology and mechanical properties.

Scaffold	Fiber diameter (nm)		Degree of alignment (%)		Ultimate strength (MPa)	Young’s modulus (MPa)
	Before crosslinking	After crosslinking	Before crosslinking	After crosslinking	After crosslinking	
Gel	603 ± 100	724 ± 146	91 ± 8	87 ± 12	0.17 ± 0.1	0.19 ± 0.05
Gel/pCelS	662 ± 134	1201 ± 138*	86 ± 13	89 ± 10	0.19 ± 0.08	0.22 ± 0.06
Gel/fCelS	681 ± 126	1227 ± 215*	89 ± 10	89 ± 10	0.19 ± 0.11	0.26 ± 0.04
Gel/PCL	683 ± 119	752 ± 89	94 ± 5	88 ± 12	1.31 ± 0.43**	1.3 ± 0.33**
Gel/PCL/pCelS	659 ± 138	975 ± 176*	93 ± 7	86 ± 13	1.89 ± 0.46**	1.4 ± 0.26**
Gel/PCL/fCelS	652 ± 144	1096 ± 130*	90 ± 9	91 ± 8	2.59 ± 0.68**	1.6 ± 0.51**

* Significantly different than Gel and Gel/PCL (*p* < 0.05).

** Significantly different than Gel, Gel/pCelS and Gel/fCelS(*p* < 0.05).

## Data Availability

All data that support the findings of this study are included within the article (and any supplementary files). Supplementary Information available at https://doi.org/10.1088/1741-2552/ae98b7/data1.

## References

[1] Yip PK and Malaspina A 2012 Spinal cord trauma and the molecular point of no return Mol. Neurodegener. 7 6 10.1186/1750-1326-7-6 PMC3299607 22315999

[2] Ahuja CS, Nori S, Tetreault L, Wilson J, Kwon B, Harrop J, Choi D and Fehlings MG 2017 Traumatic spinal cord injury-repair and regeneration Neurosurgery 80 S9–S22 10.1093/neuros/nyw080 28350947

[3] Noristani HN 2022 Intrinsic regulation of axon regeneration after spinal cord injury: recent advances and remaining challenges Exp. Neurol. 357 114198 10.1016/j.expneurol.2022.114198 35944658

[4] Huebner EA and Strittmatter SM 2009 Axon regeneration in the peripheral and central nervous systems Results Probl. Cell Differ. 48 339–51 10.1007/400_2009_19 PMC2846285 19582408

[5] Roy A, Pathak Z and Kumar H 2021 Strategies to neutralize RhoA/ROCK pathway after spinal cord injury Exp. Neurol. 343 113794 10.1016/j.expneurol.2021.113794 34166685

[6] Bhattacharyya S, Dinda A, Vishnubhatla S, Anwar MF and Jain S 2021 A combinatorial approach to modulate microenvironment toward regeneration and repair after spinal cord injury in rats Neurosci. Lett. 741 135500 10.1016/j.neulet.2020.135500 33197520

[7] Nelson DW and Gilbert RJ 2021 Extracellular matrix-mimetic hydrogels for treating neural tissue injury: a focus on fibrin, hyaluronic acid, and elastin-like polypeptide hydrogels Adv. Healthcare Mater. 10 2101329 10.1002/adhm.202101329 PMC8599642 34494398

[8] Bradbury EJ, Moon LD, Popat RJ, King VR, Bennett GS, Patel PN, Fawcett JW and McMahon SB 2002 Chondroitinase ABC promotes functional recovery after spinal cord injury Nature 416 636–40 10.1038/416636a 11948352

[9] Tully SE, Mabon R, Gama CI, Tsai SM, Liu X and Hsieh-Wilson LC 2004 A chondroitin sulfate small molecule that stimulates neuronal growth J. Am. Chem. Soc. 126 7736–7 10.1021/ja0484045 15212495

[10] Lin R, Rosahl TW, Whiting PJ, Fawcett JW and Kwok JC 2011 6-Sulphated chondroitins have a positive influence on axonal regeneration PLoS One 6 e21499 10.1371/journal.pone.0021499 PMC3128591 21747937

[11] Swarup VP, Hsiao TW, Zhang J, Prestwich GD, Kuberan B and Hlady V 2013 Exploiting differential surface display of chondroitin sulfate variants for directing neuronal outgrowth J. Am. Chem. Soc. 135 13488–94 10.1021/ja4056728 PMC3998822 23947484

[12] Wang H 2008 Chondroitin-4-sulfation negatively regulates axonal guidance and growth J. Cell Sci. 121 3083–91 10.1242/jcs.032649 PMC2562295 18768934

[13] Brown JM 2012 A sulfated carbohydrate epitope inhibits axon regeneration after injury Proc. Natl Acad. Sci. USA 109 4768–73 10.1073/pnas.1121318109 PMC3323996 22411830

[14] Schaub NJ, Johnson CD, Cooper B and Gilbert RJ 2016 Electrospun fibers for spinal cord injury research and regeneration J. Neurotrauma 33 1405–15 10.1089/neu.2015.4165 PMC4971409 26650778

[15] Menezes R, Hashemi S, Vincent R, Collins G, Meyer J, Foston M and Arinzeh TL 2019 Investigation of glycosaminoglycan mimetic scaffolds for neurite growth Acta Biomater. 90 169–78 10.1016/j.actbio.2019.03.024 30878449

[16] Nomura H, Tator CH and Shoichet MS 2006 Bioengineered strategies for spinal cord repair J. Neurotrauma 23 496–507 10.1089/neu.2006.23.496 16629632

[17] Fortun J, Hill CE and Bunge MB 2009 Combinatorial strategies with Schwann cell transplantation to improve repair of the injured spinal cord Neurosci. Lett. 456 124–32 10.1016/j.neulet.2008.08.092 PMC4809048 19429147

[18] Anderson KD 2017 Safety of autologous human Schwann cell transplantation in subacute thoracic spinal cord injury J. Neurotrauma 34 2950–63 10.1089/neu.2016.4895 28225648

[19] Gamez Sazo RE, Maenaka K, Gu W, Wood PM and Bunge MB 2012 Fabrication of growth factor- and extracellular matrix-loaded, gelatin-based scaffolds and their biocompatibility with Schwann cells and dorsal root ganglia Biomaterials 33 8529–39 10.1016/j.biomaterials.2012.07.028 PMC3521512 22906605

[20] Callizot N, Combes M, Steinschneider R and Poindron P 2011 A new long term *in vitro* model of myelination Exp. Cell Res. 317 2374–83 10.1016/j.yexcr.2011.07.002 21777582

[21] Lopez-Verrilli MA, Picou F and Court FA 2013 Schwann cell-derived exosomes enhance axonal regeneration in the peripheral nervous system Glia 61 1795–806 10.1002/glia.22558 24038411

[22] López-Leal R, Diaz P and Court FA 2018 *In vitro* analysis of the role of Schwann cells on axonal degeneration and regeneration using sensory neurons from dorsal root ganglia Methods Mol. Biol. 1739 255–67 10.1007/978-1-4939-7649-2_16 29546712

[23] Qiu J, Cafferty WB, McMahon SB and Thompson SW 2005 Conditioning injury-induced spinal axon regeneration requires signal transducer and activator of transcription 3 activation J. Neurosci. 25 1645–53 10.1523/JNEUROSCI.3269-04.2005 PMC6725934 15716400

[24] Aldskogius H and Kozlova EN 2021 Dorsal root injury-A model for exploring pathophysiology and therapeutic strategies in spinal cord injury Cells 10 2185 10.3390/cells10092185 PMC8470715 34571835

[25] Costa AC 2025 Axon-specific microtubule regulation drives asymmetric regeneration of sensory neuron axons Elife 13 RP104069 10.7554/eLife.104069 PMC11850000 39992313

[26] Neumann S and Woolf CJ 1999 Regeneration of dorsal column fibers into and beyond the lesion site following adult spinal cord injury Neuron 23 83–91 10.1016/s0896-6273(00)80755-2 10402195

[27] Huang GP, Shanmugasundaram S, Masih P, Pandya D, Amara S, Collins G and Arinzeh TL 2015 An investigation of common crosslinking agents on the stability of electrospun collagen scaffolds J. Biomed. Mater. Res. A 103 762–71 10.1002/jbm.a.35222 24828818

[28] Lee YS, Collins G and Arinzeh TL 2011 Neurite extension of primary neurons on electrospun piezoelectric scaffolds Acta Biomater. 7 3877–86 10.1016/j.actbio.2011.07.013 21810489

[29] Menezes R and Arinzeh TL 2020 Comparative study of electrospun scaffolds containing native GAGs and a GAG mimetic for human mesenchymal stem cell chondrogenesis Ann. Biomed. Eng. 48 2040–52 10.1007/s10439-020-02499-9 32285342

[30] Menezes R, Sherman L, Rameshwar P and Arinzeh TL 2023 Scaffolds containing GAG-mimetic cellulose sulfate promote TGF-beta interaction and MSC Chondrogenesis over native GAGs J. Biomed. Mater. Res. A 111 1135–50 10.1002/jbm.a.37496 PMC10277227 36708060

[31] Wu S, Chen MS, Maurel P, Lee YS, Bunge MB and Arinzeh TL 2018 Aligned fibrous PVDF-TrFE scaffolds with Schwann cells support neurite extension and myelination *in vitro* J. Neural Eng. 15 056010 10.1088/1741-2552/aac77f PMC6125183 29794323

[32] Maurel P 2018 Preparation of neonatal rat Schwann cells and embryonic dorsal root ganglia neurons for *in vitro* myelination studies Methods Mol. Biol. 1739 17–37 10.1007/978-1-4939-7649-2_2 29546698

[33] Longas MO and Breitweiser KO 1991 Sulfate composition of glycosaminoglycans determined by infrared spectroscopy Anal. Biochem. 192 193–6 10.1016/0003-2697(91)90205-8 1904690

[34] Goldberg JL 2003 How does an axon grow? Genes Dev. 17 941–58 10.1101/gad.1062303 12704078

[35] Myers JP, Santiago-medina M and Gomez TM 2011 Regulation of axonal outgrowth and pathfinding by integrin-ECM interactions Dev. Neurobiol. 71 901–23 10.1002/dneu.20931 PMC3192254 21714101

[36] Melrose J 2025 Glycosaminoglycans, instructive biomolecules that regulate cellular activity and synaptic neuronal control of specific tissue functional properties Int. J. Mol. Sci. 26 2554 10.3390/ijms26062554 PMC11942259 40141196

[37] Kanato Y, Ono S, Kitajima K and Sato C 2009 Complex formation of a brain-derived neurotrophic factor and glycosaminoglycans Biosci. Biotechnol. Biochem. 73 2735–41 10.1271/bbb.90637 19966464

[38] Li Y, Sun C, Yates EA, Jiang C, Wilkinson MC and Fernig DG 2016 Heparin binding preference and structures in the fibroblast growth factor family parallel their evolutionary diversification Open Biol. 6 150275 10.1098/rsob.150275 PMC4821243 27030175

[39] Brushart TM, Aspalter M, Griffin JW, Redett R, Hameed H, Zhou C, Wright M, Vyas A and Höke A 2013 Schwann cell phenotype is regulated by axon modality and central-peripheral location, and persists *in vitro* Exp. Neurol. 247 272–81 10.1016/j.expneurol.2013.05.007 PMC3742710 23707299

[40] Bunge RP 1994 The role of the Schwann cell in trophic support and regeneration J. Neurol. 242 S19–21 10.1007/BF00939235 7699403

[41] Anselme K, Ploux L and Ponche A 2010 Cell/material interfaces: influence of surface chemistry and surface topography on cell adhesion J. Adhes. Sci. Technol. 24 831–52

[42] Johnson KM, Milner R and Crocker SJ 2015 Extracellular matrix composition determines astrocyte responses to mechanical and inflammatory stimuli Neurosci. Lett. 600 104–9 10.1016/j.neulet.2015.06.013 PMC4530625 26067407

[43] Rocha DN, Ferraz-Nogueira JP, Barrias CC, Relvas JB and Pego AP 2015 Extracellular environment contribution to astrogliosis-lessons learned from a tissue engineered 3D model of the glial scar Front. Cell. Neurosci. 9 377 10.3389/fncel.2015.00377 PMC4586948 26483632

[44] Puschmann TB, Zanden C, De Pablo Y, Kirchhoff F, Pekna M, Liu J and Pekny M 2013 Bioactive 3D cell culture system minimizes cellular stress and maintains the *in vivo*-like morphological complexity of astroglial cells Glia 61 432–40 10.1002/glia.22446 23292921

[45] Zhou B, Zuo YX and Jiang RT 2019 Astrocyte morphology: diversity, plasticity, and role in neurological diseases CNS Neurosci. Ther. 25 665–73 10.1111/cns.13123 PMC6515705 30929313

[46] Wiese S, Karus M and Faissner A 2012 Astrocytes as a source for extracellular matrix molecules and cytokines Front. Pharmacol. 3 120 10.3389/fphar.2012.00120 PMC3382726 22740833

[47] Puhl DL, Funnell JL, Nelson DW, Gottipati MK and Gilbert RJ 2021 Electrospun fiber scaffolds for engineering glial cell behavior to promote neural regeneration Bioengineering 8 4 10.3390/bioengineering8010004 PMC7823609 33383759

[48] Maurel P and Salzer JL 2000 Axonal regulation of Schwann cell proliferation and survival and the initial events of myelination requires PI 3-kinase activity J. Neurosci. 20 4635–45 10.1523/JNEUROSCI.20-12-04635.2000 PMC6772460 10844033

[49] Grima B, Zelenika D and Pessac B 1992 A novel transcript overlapping the myelin basic protein gene J. Neurochem. 59 2318–23 10.1111/j.1471-4159.1992.tb10126.x 1279125

[50] Salzer JL and Zalc B 2016 Myelination Curr. Biol. 26 R971–r5 10.1016/j.cub.2016.07.074 27780071

[51] Bosch-queralt M, Fledrich R and Stassart RM 2023 Schwann cell functions in peripheral nerve development and repair Neurobiol. Dis. 176 105952 10.1016/j.nbd.2022.105952 36493976

[52] Fu H, Hu D, Chen J, Wang Q, Zhang Y, Qi C and Yu T 2022 Repair of the injured spinal cord by Schwann cell transplantation Front. Neurosci. 16 800513 10.3389/fnins.2022.800513 PMC8891437 35250447

[53] Eshed-Eisenbach Y, Gordon A, Sukhanov N and Peles E 2016 Specific inhibition of secreted NRG1 types III by heparin enhances Schwann cell myelination Glia 64 1227–34 10.1002/glia.22995 27143444

[54] Nagayasu T, Miyata S, Hayashi N, Takano R, Kariya Y and Kamei K 2005 Heparin structures in FGF-2-dependent morphological transformation of astrocytes J. Biomed. Mater. Res. A 74 374–80 10.1002/jbm.a.30338 15973728

[55] Baiguera S, Del Gaudio C, Lucatelli E, Kuevda E, Boieri M, Mazzanti B, Bianco A and Macchiarini P 2014 Electrospun gelatin scaffolds incorporating rat decellularized brain extracellular matrix for neural tissue engineering Biomaterials 35 1205–14 10.1016/j.biomaterials.2013.10.060 24215734

