# Supplementary material for: Aligned fibrous scaffolds containing glycosaminoglycan mimetics enhance Schwann cell-supported axon growth and myelination
Source: J Neural Eng. Author manuscript; Available in PMC 2026 Oct 6. (PMC13641151; doi:10.1088/1741-2552/ae98b7)
Supplement: Supplementary file 1 [file NIHMS2211947-supplement-Supplemental_Material.docx]

**Supplemental Information**

*S.1. Materials*

Gelatin type B from bovine skin (Millipore Sigma, MO, USA); polycaprolactone (PCL) 80,000 Mn (Millipore Sigma); 2,2,2-Trifluoroethanol (TFE) (Millipore Sigma); acetic Acid (Millipore Sigma); DI water (Milli-Q); partially sulfated cellulose (pCelS, prepared as published [57], with 0.54 sulfate group per monosaccharide; MW: 290 kDa) and fully sulfate cellulose (fCelS, with 2.3 sulfate group per monosaccharide, MW: 1,400 kDa, Dextran Products Limited, Ontario, Canada); 100% ethanol (Thermofisher, MA, USA), N- (3-Dimethylaminopropyl)-N’-ethyl carbodiimide (EDC) (Novabiochem, Merck KGaA, Germany), N- hydroxysulfosuccinimide (NHS) (Millipore Sigma); sodium phosphate dibasic (Na_2_HPO_4_) (Millipore Sigma). Tissue culture plates (Corning, NY, USA); Ultra-low attachment multi-well plate (Cornin); Glutamax 100X (Gibco, MD, NY, USA); Trypsin-EDTA 0.25% (Gibco); Penicillin-Streptomycin 10,000 U/ml (Gibco); Gentamicin 50mg/ml (Gibco); L-ascorbic acid (Millipore Sigma); Minimum Essential Medium (MEM) low glucose without L-glutamine (Gibco); Dulbecco's Modified Eagle Medium (DMEM) high glucose containing 4mM L-glutamine (Gibco); Fetal bovine serum (Hyclone, UT, USA); Forskolin (Millipore Sigma); Bovine pituitary extract (Biomedical Technologies, NJ, USA); Heregulin (R&D System, MN, USA); Leibovitz’s L-15 media (Gibco); D-D-(+)-Glucose (Millipore Sigma); nerve growth factor 7S (Gibco); nerve growth factor 2.5S (Bio-Rad, PA, USA); 1X Dulbecco’s phosphate-buffered saline (DPBS) without calcium and magnesium (Gibco); Heat Inactivated Fetal Bovine Serum (HI-FBS) (Gibco); bovine serum albumin (BSA) (HyClone); Reduced Growth Factor Matrigel (Corning); collagen type I, rat (Corning); Poly-L-lysine hydrobromide, (PLL) MW 30,000-70,000 (Millipore Sigma); bacid (Millipore Sigma); borax anhydrous (Millipore Sigma); Quant-iT dsDNA Picogreen Assay Kit (Invitrogen, CA, USA); Papain protease (Millipore Sigma); Triton X-100 (Millipore Sigma); Paraformaldehyde (Millipore Sigma); cysteine (MilliporeSigma); disodium ethylenediaminetetraacetate dihydrate (Na2EDTA.2H2O) (MilliporeSigma); Mitotic inhibitor (5-fluoro-2’-deoxyuridine/Uridin) (Millipore Sigma); ammonium hydroxide (MilliporeSigma); NT-3 ELISA kit (R&D System); BDNF ELISA kit (R&D System); 4',6-diamidino-2-phenylindole (Dapi) (Invitrogen); Micro BCA protein assay kit (Thermofisher); rabbit serum (Millipore Sigma); donkey serum (Millipore Sigma); goat serum (MilliporeSigma); mouse anti-Myelin Basic Protein (MBP) (Biolegend, MA, USA); Rabbit anti-MBP (1:200) (MilliporeSigma); rabbit anti-neurofilament primary (Millipore Sigma); mouse anti-Caspr primary antibody (Antibodies Inc.); Myelin Basic Protein (MBP) ELISA kit (Anitbodies-Online, PA, USA); Glyceraldehyde 3-phosphate dehydrogenase (GAPDH) ELISA kit (Antibodies-Online); phenylmethylsulfonyl fluoride (PMSF) (Thermofisher); Halt protease inhibitor cocktail, EDTA-Free 100x (Thermofisher); Cell Extraction Buffer (Thermofisher); Dimethylsulfoxide (DMSO) (Thermofisher).

S.2. SC, DRG and Ast isolation and culture media

SCs isolated from adult rat sciatic nerves were provided by the University of Miami as previously reported [32]. Transfected SCs expressing enhanced green fluorescent protein (eGFP-SCs) or non-transfected SCs were used at passage 2. Sprague Dawley rats were purchased from Charles River Laboratories International Inc. (Wilmington, MA). Rats were housed and procedures performed at Rutgers, The State University of New Jersey, in accordance with an animal protocol approved by Rutgers University IACUC. Asts were isolated from 6-7 weeks old rat spinal cords, and embryonic rat dorsal root ganglions (DRGs) were isolated from E-16 rat embryos pregnant rats. DRGs were used in this study to evaluate axon growth since they are commonly used as a model to evaluate growth of axons on biomaterials [19, 20]. Lumbar DRGs were isolated following a previously reported protocol [33]. Asts were isolated from cleaned spinal cord tissue free from meninges and dissociated in Neuron Isolation Enzyme containing Papain following the manufacturer protocol. Cells were seeded onto poly-L-lysine-coated (PLL) culture plates and maintained in Ast media for one week. The culture was shaken for 2 h at 200 rpm using an orbital shaker to remove non-adherent microglia cells and oligodendrocytes. When confluent, the culture was incubated in TrypLE Express Enzyme for 10 min at 37^o^C, centrifuged at 1900 rpm for 8 min, and resuspended in FBS free media. The cell suspension was plated onto a non-treated culture plate for 5 min to remove remaining microglia. The floating Asts were centrifuged again, re-plated on PLL coted plates, and maintained in Ast media until use. Ast purity (99%) was confirmed by counting the number of glial fibrillary acidic protein (GFAP) positive cells over the total number of nuclei (data not shown).

The following medium were prepared. SC media: DMEM containing 2 μM forskolin 2.5 μM heregulin 2% Bovine pituitary extract, 10% HI-FBS, and 0.1% Gentamicin. Ast media: MEM containing 10% HI-FBS and 1% penicillin-streptomycin. DRG media (DN): neurobasal medium containing 50 ng/ml of 2.5S-NGF, 2 % B-27 50x, 0.05 mg/ml of Gentamicin, 0.08 % D-glucose, and 1 mM Glutamax. DRG explant growth media (NB): neurobasal medium containing 50 ng/ml of 7S-NGF, 2% B-27 50X, 1 % penicillin-streptomycin 0.08 % D-glucose, and 1 mM Glutamax. DRG explant purification media (NBF): NB media containing the mixture of 20 µM mitotic inhibitor 5-fluoro-2’-deoxyuridine (FdUr) and Uridin. Co-culture media for neurite extension (CN): MEM containing 10% HI-FBS, 50 ng/ml 7S-NGF, 4% D-Glucose, 1 mM Glutamax, and 1 % penicillin-streptomycin. Co-culture media for myelination (CM): MEM containing 10% HI-FBS, 50 ng/ml 2.5S-NGF, 0.05 mg/ml of Gentamicin, 0.08 % D-glucose, and 1 mM Glutamax.

S.3. Immunostaining procedure

For SCs, Asts, and axon growth, all samples were fixed using 4% paraformaldehyde (PFA) for 20 min at RT, and permeabilized by 0.1% Triton-X100 in a blocking solution containing 2% BSA and 10% normal goat and/or donkey serum in DPBS for 1 h at RT. All myelination samples were fixed in 1 or 4% PFA for MBP/Caspr or MBP/NF respectively for 20 minutes at RT. Samples were permeabilized by ice-cold methanol for 25 min and then incubated in the blocking solution containing 0.3% Triton-X100, 2% BSA and 10% normal donkey and goat serum in DPBS for 1 h at RT. All samples were incubated with the appropriate primary antibody overnight at 4 ^o^C, and then rinsed with DPBS and incubated with the appropriate secondary antibodies for 1 – 1.5 h. The primary antibodies were rabbit anti-NF (1:1000); mouse anti-GFAP (1:200); mouse anti-MBP (1:200); rabbit anti-MBP (1:100); mouse anti-Caspr antibody (1:200). The secondary antibodies were Alexa Fluor 488 conjugated goat anti-rabbit (1:500); Alexa Fluor 555 conjugated donkey anti-mouse (1:400); Alexa Fluor 488 conjugated goat anti-mouse (1:2000); Alexa Fluor 594 donkey anti-rabbit (1:500); Alexa Fluor 594 goat anti-rabbit (1:800). The nucleus was stained with DAPI for 1 min prior to imaging.

**Fig. S1.** Structure of A) Chondroitin sulfate-C (CS-C) with sulfate group at C-6. B) partially sulfated cellulose (pCelS). C) fully sulfated cellulose (fCelS).

**Fig. S2.** Swelling behavior of aligned fibrous Gel/PCL scaffolds with varying weight % of Gel to PCL, following immersion in 1X DPBS for 24 hours ; n=6 per group; *p<0.05. Column scatter plot where the bar and error bars represent the Mean ± S.D..

Table S1. Mechanical properties of aligned fibrous Gel/PCL scaffolds having varying weight% of Gel to PCL.

| Gelatin (Gel)% | Polycaprolactone (PCL) % | Young’s Modulus (MPa) | Ultimate Strength (MPa) |
| --- | --- | --- | --- |
| 100 | 0 | 0.12 (± 0.03) | 0.15(± 0.04) |
| 95 | 5 | 0.73 (± 0.18) | 0.33(± 0.10) |
| 90 | 10 | 0.90 (± 0.20) | 0.85± 0.62) |
| 85 | 15 | 1.12 (± 0.20) | 0.97 (± 0.43) |
| 80 | 20 | **1.39(± 0.27)**** | **2.24(± 0.74)**** |

**Significantly different than Gel scaffold alone (p<0.05); n=6 per group. Values are Mean ± S.D.

**A**

**B**

Absorbance

| Wavenumber (cm-1) | Gel | Gel/pCelS | Gel/fCelS |
| --- | --- | --- | --- |
| 4000 | 0.129988 | 0.357461 | 0.536542 |
| 3999 | 0.129988 | 0.357049 | 0.536898 |
| 3998 | 0.129383 | 0.356636 | 0.536898 |
| 3997 | 0.128779 | 0.356224 | 0.537255 |
| 3996 | 0.128174 | 0.355812 | 0.536898 |
| 3995 | 0.128174 | 0.355812 | 0.536542 |
| 3994 | 0.128174 | 0.356224 | 0.535829 |
| 3993 | 0.128174 | 0.356636 | 0.535472 |
| 3992 | 0.128779 | 0.356636 | 0.535472 |
| 3991 | 0.128779 | 0.356636 | 0.535829 |
| 3990 | 0.128779 | 0.356224 | 0.536185 |
| 3989 | 0.128779 | 0.356224 | 0.536542 |
| 3988 | 0.128779 | 0.355812 | 0.536542 |
| 3987 | 0.129383 | 0.3554 | 0.536185 |
| 3986 | 0.129988 | 0.3554 | 0.535829 |
| 3985 | 0.129383 | 0.3554 | 0.535472 |
| 3984 | 0.129383 | 0.355812 | 0.535116 |
| 3983 | 0.129383 | 0.356224 | 0.535116 |
| 3982 | 0.129383 | 0.355812 | 0.535116 |
| 3981 | 0.129383 | 0.355812 | 0.535472 |
| 3980 | 0.129383 | 0.355812 | 0.535829 |
| 3979 | 0.129383 | 0.356224 | 0.535829 |
| 3978 | 0.129383 | 0.356636 | 0.535829 |
| 3977 | 0.129988 | 0.356636 | 0.535472 |
| 3976 | 0.129988 | 0.356224 | 0.535472 |
| 3975 | 0.129988 | 0.3554 | 0.535472 |
| 3974 | 0.129383 | 0.3554 | 0.535829 |
| 3973 | 0.128779 | 0.354988 | 0.536185 |
| 3972 | 0.12757 | 0.3554 | 0.536185 |
| 3971 | 0.12757 | 0.3554 | 0.536185 |
| 3970 | 0.126965 | 0.3554 | 0.535829 |
| 3969 | 0.126965 | 0.3554 | 0.535829 |
| 3968 | 0.126965 | 0.354988 | 0.536185 |
| 3967 | 0.126965 | 0.354988 | 0.536185 |
| 3966 | 0.126965 | 0.354988 | 0.536542 |
| 3965 | 0.12757 | 0.354575 | 0.536542 |
| 3964 | 0.12757 | 0.354575 | 0.536542 |
| 3963 | 0.12757 | 0.354163 | 0.536185 |
| 3962 | 0.12757 | 0.354163 | 0.535829 |
| 3961 | 0.128174 | 0.353751 | 0.535116 |
| 3960 | 0.128779 | 0.353751 | 0.534403 |
| 3959 | 0.129383 | 0.353751 | 0.534046 |
| 3958 | 0.129383 | 0.354163 | 0.534046 |
| 3957 | 0.129383 | 0.354575 | 0.534046 |
| 3956 | 0.129383 | 0.354575 | 0.534046 |
| 3955 | 0.128779 | 0.354575 | 0.534403 |
| 3954 | 0.128779 | 0.354163 | 0.534759 |
| 3953 | 0.128779 | 0.353751 | 0.535116 |
| 3952 | 0.128779 | 0.353751 | 0.535472 |
| 3951 | 0.129383 | 0.353751 | 0.535472 |
| 3950 | 0.130593 | 0.353751 | 0.535116 |
| 3949 | 0.131802 | 0.353751 | 0.534759 |
| 3948 | 0.131802 | 0.353339 | 0.534403 |
| 3947 | 0.131802 | 0.352927 | 0.534403 |
| 3946 | 0.131197 | 0.352514 | 0.534759 |
| 3945 | 0.131197 | 0.352514 | 0.535116 |
| 3944 | 0.131197 | 0.352514 | 0.535116 |
| 3943 | 0.130593 | 0.353339 | 0.535116 |
| 3942 | 0.130593 | 0.353751 | 0.534759 |
| 3941 | 0.130593 | 0.353751 | 0.534046 |
| 3940 | 0.130593 | 0.353339 | 0.53369 |
| 3939 | 0.131197 | 0.352927 | 0.533333 |
| 3938 | 0.131197 | 0.352514 | 0.533333 |
| 3937 | 0.130593 | 0.352102 | 0.533333 |
| 3936 | 0.129988 | 0.352102 | 0.533333 |
| 3935 | 0.129988 | 0.352514 | 0.533333 |
| 3934 | 0.129988 | 0.352927 | 0.533333 |
| 3933 | 0.129383 | 0.353339 | 0.533333 |
| 3932 | 0.128174 | 0.353339 | 0.533333 |
| 3931 | 0.128174 | 0.353339 | 0.533333 |
| 3930 | 0.129383 | 0.353339 | 0.533333 |
| 3929 | 0.129988 | 0.353751 | 0.533333 |
| 3928 | 0.130593 | 0.353751 | 0.532977 |
| 3927 | 0.130593 | 0.353751 | 0.532977 |
| 3926 | 0.129988 | 0.353751 | 0.532977 |
| 3925 | 0.128779 | 0.353751 | 0.533333 |
| 3924 | 0.128174 | 0.353339 | 0.53369 |
| 3923 | 0.128174 | 0.352927 | 0.534046 |
| 3922 | 0.128779 | 0.352514 | 0.534759 |
| 3921 | 0.129988 | 0.352514 | 0.534759 |
| 3920 | 0.129988 | 0.352927 | 0.534759 |
| 3919 | 0.129383 | 0.353751 | 0.534403 |
| 3918 | 0.129383 | 0.353751 | 0.534046 |
| 3917 | 0.130593 | 0.353339 | 0.533333 |
| 3916 | 0.131197 | 0.352927 | 0.532977 |
| 3915 | 0.131197 | 0.352514 | 0.53262 |
| 3914 | 0.130593 | 0.352102 | 0.53262 |
| 3913 | 0.129383 | 0.35169 | 0.53262 |
| 3912 | 0.12757 | 0.35169 | 0.532977 |
| 3911 | 0.125756 | 0.35169 | 0.532977 |
| 3910 | 0.124547 | 0.35169 | 0.533333 |
| 3909 | 0.124547 | 0.352102 | 0.53369 |
| 3908 | 0.125151 | 0.352514 | 0.534046 |
| 3907 | 0.126965 | 0.352927 | 0.534046 |
| 3906 | 0.128174 | 0.352927 | 0.534046 |
| 3905 | 0.129988 | 0.352927 | 0.534046 |
| 3904 | 0.131802 | 0.352514 | 0.534403 |
| 3903 | 0.133011 | 0.352102 | 0.534403 |
| 3902 | 0.134825 | 0.352102 | 0.534403 |
| 3901 | 0.136034 | 0.352102 | 0.534046 |
| 3900 | 0.137243 | 0.352102 | 0.533333 |
| 3899 | 0.136034 | 0.351278 | 0.532977 |
| 3898 | 0.13422 | 0.350866 | 0.532977 |
| 3897 | 0.132406 | 0.350866 | 0.532977 |
| 3896 | 0.131802 | 0.351278 | 0.533333 |
| 3895 | 0.131197 | 0.352102 | 0.532977 |
| 3894 | 0.130593 | 0.352927 | 0.532977 |
| 3893 | 0.129988 | 0.352927 | 0.53262 |
| 3892 | 0.128779 | 0.352514 | 0.53262 |
| 3891 | 0.12757 | 0.352102 | 0.532977 |
| 3890 | 0.126965 | 0.352102 | 0.533333 |
| 3889 | 0.126965 | 0.352102 | 0.533333 |
| 3888 | 0.128174 | 0.352102 | 0.533333 |
| 3887 | 0.129988 | 0.352102 | 0.532977 |
| 3886 | 0.129383 | 0.35169 | 0.53262 |
| 3885 | 0.128174 | 0.35169 | 0.532264 |
| 3884 | 0.128174 | 0.351278 | 0.531907 |
| 3883 | 0.131197 | 0.350866 | 0.531551 |
| 3882 | 0.135429 | 0.349629 | 0.531194 |
| 3881 | 0.136638 | 0.348392 | 0.530481 |
| 3880 | 0.134825 | 0.34798 | 0.529412 |
| 3879 | 0.131802 | 0.34798 | 0.529055 |
| 3878 | 0.130593 | 0.348392 | 0.529055 |
| 3877 | 0.131197 | 0.348805 | 0.529412 |
| 3876 | 0.132406 | 0.348805 | 0.530481 |
| 3875 | 0.132406 | 0.348805 | 0.531194 |
| 3874 | 0.130593 | 0.349217 | 0.531907 |
| 3873 | 0.129383 | 0.350041 | 0.532264 |
| 3872 | 0.130593 | 0.350453 | 0.532264 |
| 3871 | 0.132406 | 0.350453 | 0.531907 |
| 3870 | 0.133011 | 0.350453 | 0.531551 |
| 3869 | 0.131197 | 0.350453 | 0.531194 |
| 3868 | 0.129383 | 0.350453 | 0.531194 |
| 3867 | 0.130593 | 0.350453 | 0.531551 |
| 3866 | 0.133011 | 0.350453 | 0.531194 |
| 3865 | 0.133011 | 0.350041 | 0.530838 |
| 3864 | 0.131197 | 0.350041 | 0.530125 |
| 3863 | 0.129988 | 0.349629 | 0.529768 |
| 3862 | 0.129988 | 0.349629 | 0.530125 |
| 3861 | 0.130593 | 0.349217 | 0.530481 |
| 3860 | 0.129383 | 0.348805 | 0.530838 |
| 3859 | 0.128174 | 0.348392 | 0.531194 |
| 3858 | 0.12636 | 0.348392 | 0.530838 |
| 3857 | 0.125151 | 0.34798 | 0.530838 |
| 3856 | 0.125151 | 0.34798 | 0.531194 |
| 3855 | 0.125756 | 0.347568 | 0.531907 |
| 3854 | 0.12636 | 0.347156 | 0.53262 |
| 3853 | 0.12636 | 0.347156 | 0.53369 |
| 3852 | 0.125756 | 0.347156 | 0.534403 |
| 3851 | 0.126965 | 0.347156 | 0.534403 |
| 3850 | 0.129383 | 0.347156 | 0.53369 |
| 3849 | 0.131197 | 0.346744 | 0.532264 |
| 3848 | 0.129988 | 0.346331 | 0.531194 |
| 3847 | 0.12757 | 0.345919 | 0.530125 |
| 3846 | 0.125151 | 0.345507 | 0.529768 |
| 3845 | 0.123337 | 0.345095 | 0.529412 |
| 3844 | 0.123337 | 0.345095 | 0.528699 |
| 3843 | 0.123337 | 0.345095 | 0.527986 |
| 3842 | 0.124547 | 0.345507 | 0.527273 |
| 3841 | 0.126965 | 0.345507 | 0.527629 |
| 3840 | 0.129383 | 0.345507 | 0.528699 |
| 3839 | 0.130593 | 0.345095 | 0.529768 |
| 3838 | 0.131197 | 0.345095 | 0.530481 |
| 3837 | 0.130593 | 0.344683 | 0.530481 |
| 3836 | 0.129988 | 0.344683 | 0.530125 |
| 3835 | 0.129988 | 0.345095 | 0.530125 |
| 3834 | 0.130593 | 0.345095 | 0.530481 |
| 3833 | 0.130593 | 0.345507 | 0.531194 |
| 3832 | 0.129988 | 0.345095 | 0.531551 |
| 3831 | 0.128779 | 0.345095 | 0.531194 |
| 3830 | 0.128779 | 0.34427 | 0.530125 |
| 3829 | 0.129383 | 0.343858 | 0.528699 |
| 3828 | 0.130593 | 0.343034 | 0.527629 |
| 3827 | 0.129988 | 0.343034 | 0.526916 |
| 3826 | 0.128174 | 0.343446 | 0.526916 |
| 3825 | 0.125151 | 0.343858 | 0.527273 |
| 3824 | 0.125151 | 0.344683 | 0.527986 |
| 3823 | 0.126965 | 0.344683 | 0.528699 |
| 3822 | 0.128174 | 0.34427 | 0.529412 |
| 3821 | 0.12757 | 0.34427 | 0.529768 |
| 3820 | 0.125756 | 0.344683 | 0.529768 |
| 3819 | 0.125151 | 0.345095 | 0.529412 |
| 3818 | 0.12757 | 0.344683 | 0.529412 |
| 3817 | 0.129383 | 0.34427 | 0.529412 |
| 3816 | 0.128174 | 0.343858 | 0.529055 |
| 3815 | 0.125151 | 0.343446 | 0.528699 |
| 3814 | 0.123337 | 0.343446 | 0.527629 |
| 3813 | 0.123942 | 0.343446 | 0.52656 |
| 3812 | 0.125151 | 0.343446 | 0.525847 |
| 3811 | 0.123942 | 0.343034 | 0.525134 |
| 3810 | 0.121524 | 0.342622 | 0.525134 |
| 3809 | 0.120314 | 0.341797 | 0.525847 |
| 3808 | 0.120919 | 0.340973 | 0.52656 |
| 3807 | 0.122128 | 0.340561 | 0.527629 |
| 3806 | 0.122733 | 0.340561 | 0.528342 |
| 3805 | 0.123942 | 0.340973 | 0.528699 |
| 3804 | 0.12636 | 0.341797 | 0.528699 |
| 3803 | 0.128779 | 0.342622 | 0.528699 |
| 3802 | 0.128174 | 0.343034 | 0.528342 |
| 3801 | 0.124547 | 0.343446 | 0.527629 |

| Wavenumber (cm-1) | Gel/PCL | Gel/PCL/pCelS | Gel/PCL/fCelS | PCL |
| --- | --- | --- | --- | --- |
| 4000 | 0.029098 | -0.199784 | -0.15 | 0.213929 |
| 3999 | 0.0299653 | -0.200007 | -0.149778 | 0.213818 |
| 3998 | 0.0308326 | -0.200007 | -0.149335 | 0.213818 |
| 3997 | 0.0312663 | -0.200229 | -0.148671 | 0.213818 |
| 3996 | 0.0316999 | -0.200451 | -0.148006 | 0.213818 |
| 3995 | 0.0316999 | -0.200673 | -0.148006 | 0.213708 |
| 3994 | 0.0312663 | -0.201118 | -0.148227 | 0.213708 |
| 3993 | 0.030399 | -0.20134 | -0.148449 | 0.213708 |
| 3992 | 0.0295317 | -0.20134 | -0.148671 | 0.213708 |
| 3991 | 0.0295317 | -0.20134 | -0.148671 | 0.213929 |
| 3990 | 0.0295317 | -0.200895 | -0.148671 | 0.21415 |
| 3989 | 0.0295317 | -0.200895 | -0.148671 | 0.214371 |
| 3988 | 0.0295317 | -0.200895 | -0.148671 | 0.214482 |
| 3987 | 0.0295317 | -0.200895 | -0.148449 | 0.214482 |
| 3986 | 0.0299653 | -0.200673 | -0.148227 | 0.214592 |
| 3985 | 0.0299653 | -0.200451 | -0.148227 | 0.214592 |
| 3984 | 0.030399 | -0.200007 | -0.148227 | 0.214592 |
| 3983 | 0.0308326 | -0.199562 | -0.148227 | 0.214592 |
| 3982 | 0.030399 | -0.19934 | -0.148227 | 0.214592 |
| 3981 | 0.0299653 | -0.199118 | -0.148449 | 0.214482 |
| 3980 | 0.029098 | -0.19934 | -0.148449 | 0.21426 |
| 3979 | 0.0282307 | -0.199562 | -0.148449 | 0.213929 |
| 3978 | 0.0277971 | -0.200007 | -0.148449 | 0.213708 |
| 3977 | 0.0277971 | -0.200451 | -0.148227 | 0.213597 |
| 3976 | 0.0277971 | -0.200895 | -0.148006 | 0.213708 |
| 3975 | 0.0277971 | -0.201118 | -0.147784 | 0.213818 |
| 3974 | 0.0277971 | -0.201118 | -0.147563 | 0.213929 |
| 3973 | 0.0282307 | -0.200895 | -0.147784 | 0.213929 |
| 3972 | 0.0282307 | -0.200895 | -0.147784 | 0.213818 |
| 3971 | 0.0286644 | -0.201118 | -0.147563 | 0.213708 |
| 3970 | 0.029098 | -0.201118 | -0.147119 | 0.213708 |
| 3969 | 0.029098 | -0.200895 | -0.146676 | 0.213929 |
| 3968 | 0.029098 | -0.200451 | -0.146233 | 0.21426 |
| 3967 | 0.029098 | -0.200229 | -0.146455 | 0.214482 |
| 3966 | 0.0282307 | -0.200007 | -0.146898 | 0.214482 |
| 3965 | 0.0269297 | -0.200007 | -0.147341 | 0.214371 |
| 3964 | 0.0260624 | -0.200229 | -0.147784 | 0.21415 |
| 3963 | 0.0256288 | -0.200451 | -0.148006 | 0.213929 |
| 3962 | 0.0260624 | -0.200451 | -0.147784 | 0.213929 |
| 3961 | 0.0264961 | -0.200673 | -0.147563 | 0.213818 |
| 3960 | 0.0273634 | -0.200895 | -0.147119 | 0.213818 |
| 3959 | 0.0273634 | -0.200895 | -0.146676 | 0.213818 |
| 3958 | 0.0269297 | -0.200895 | -0.146455 | 0.213929 |
| 3957 | 0.0264961 | -0.200895 | -0.146676 | 0.214039 |
| 3956 | 0.0260624 | -0.200673 | -0.146898 | 0.21415 |
| 3955 | 0.0264961 | -0.200451 | -0.147119 | 0.21415 |
| 3954 | 0.0269297 | -0.200451 | -0.147341 | 0.213929 |
| 3953 | 0.0273634 | -0.200895 | -0.147341 | 0.213487 |
| 3952 | 0.0273634 | -0.201784 | -0.146898 | 0.213044 |
| 3951 | 0.0269297 | -0.202895 | -0.146233 | 0.212602 |
| 3950 | 0.0264961 | -0.203561 | -0.145347 | 0.212381 |
| 3949 | 0.0264961 | -0.204006 | -0.14446 | 0.212381 |
| 3948 | 0.0260624 | -0.204228 | -0.143796 | 0.212492 |
| 3947 | 0.0256288 | -0.204672 | -0.143574 | 0.212492 |
| 3946 | 0.0251951 | -0.205339 | -0.143353 | 0.212271 |
| 3945 | 0.0251951 | -0.205339 | -0.143131 | 0.21216 |
| 3944 | 0.0256288 | -0.204672 | -0.143131 | 0.212381 |
| 3943 | 0.0264961 | -0.203339 | -0.143353 | 0.212934 |
| 3942 | 0.0269297 | -0.202673 | -0.144017 | 0.213376 |
| 3941 | 0.0273634 | -0.202673 | -0.144017 | 0.213376 |
| 3940 | 0.0277971 | -0.202673 | -0.143796 | 0.213266 |
| 3939 | 0.0277971 | -0.202006 | -0.144017 | 0.213487 |
| 3938 | 0.0282307 | -0.201118 | -0.144682 | 0.213929 |
| 3937 | 0.0282307 | -0.200673 | -0.14579 | 0.214039 |
| 3936 | 0.0277971 | -0.201562 | -0.146233 | 0.213818 |
| 3935 | 0.0269297 | -0.202895 | -0.145568 | 0.213155 |
| 3934 | 0.0256288 | -0.204006 | -0.14446 | 0.212602 |
| 3933 | 0.0247615 | -0.204228 | -0.143574 | 0.212381 |
| 3932 | 0.0247615 | -0.204228 | -0.142909 | 0.212492 |
| 3931 | 0.0251951 | -0.204006 | -0.142245 | 0.212713 |
| 3930 | 0.0260624 | -0.204006 | -0.142023 | 0.212823 |
| 3929 | 0.0264961 | -0.204228 | -0.141801 | 0.212713 |
| 3928 | 0.0264961 | -0.20445 | -0.141801 | 0.212602 |
| 3927 | 0.0264961 | -0.204672 | -0.14158 | 0.212492 |
| 3926 | 0.0264961 | -0.204228 | -0.141358 | 0.212602 |
| 3925 | 0.0269297 | -0.203339 | -0.14158 | 0.212934 |
| 3924 | 0.0264961 | -0.202895 | -0.142023 | 0.212934 |
| 3923 | 0.0260624 | -0.203561 | -0.141801 | 0.212602 |
| 3922 | 0.0256288 | -0.204672 | -0.141358 | 0.21205 |
| 3921 | 0.0247615 | -0.205561 | -0.141358 | 0.211718 |
| 3920 | 0.0243278 | -0.206005 | -0.14158 | 0.211607 |
| 3919 | 0.0243278 | -0.206228 | -0.14158 | 0.211607 |
| 3918 | 0.0243278 | -0.206005 | -0.140915 | 0.211828 |
| 3917 | 0.0238942 | -0.205117 | -0.14025 | 0.212271 |
| 3916 | 0.0243278 | -0.204228 | -0.14025 | 0.212713 |
| 3915 | 0.0247615 | -0.203339 | -0.140694 | 0.212934 |
| 3914 | 0.0251951 | -0.202451 | -0.140915 | 0.213155 |
| 3913 | 0.0256288 | -0.200895 | -0.141358 | 0.213597 |
| 3912 | 0.0256288 | -0.199118 | -0.142245 | 0.21415 |
| 3911 | 0.0251951 | -0.198229 | -0.143131 | 0.214482 |
| 3910 | 0.0247615 | -0.198896 | -0.144017 | 0.214371 |
| 3909 | 0.0238942 | -0.201118 | -0.14446 | 0.213597 |
| 3908 | 0.0230269 | -0.204228 | -0.14446 | 0.21216 |
| 3907 | 0.0225932 | -0.208227 | -0.143796 | 0.210502 |
| 3906 | 0.0221596 | -0.211782 | -0.142466 | 0.208844 |
| 3905 | 0.0221596 | -0.214226 | -0.141137 | 0.207738 |
| 3904 | 0.0217259 | -0.215781 | -0.139364 | 0.207075 |
| 3903 | 0.0217259 | -0.216892 | -0.137148 | 0.206412 |
| 3902 | 0.0217259 | -0.218003 | -0.134046 | 0.205969 |
| 3901 | 0.0221596 | -0.217336 | -0.130944 | 0.206412 |
| 3900 | 0.0225932 | -0.214448 | -0.129614 | 0.207849 |
| 3899 | 0.0230269 | -0.210893 | -0.130944 | 0.209618 |
| 3898 | 0.0230269 | -0.208671 | -0.133603 | 0.210834 |
| 3897 | 0.0230269 | -0.207783 | -0.135819 | 0.211276 |
| 3896 | 0.0221596 | -0.207338 | -0.137591 | 0.211386 |
| 3895 | 0.0217259 | -0.206894 | -0.138921 | 0.211276 |
| 3894 | 0.0212923 | -0.207338 | -0.139586 | 0.211055 |
| 3893 | 0.0208586 | -0.207338 | -0.139142 | 0.210944 |
| 3892 | 0.0208586 | -0.206005 | -0.138256 | 0.211386 |
| 3891 | 0.0208586 | -0.204894 | -0.138699 | 0.211828 |
| 3890 | 0.0208586 | -0.206228 | -0.139807 | 0.211276 |
| 3889 | 0.0208586 | -0.209782 | -0.139364 | 0.209728 |
| 3888 | 0.0208586 | -0.21156 | -0.137148 | 0.208733 |
| 3887 | 0.0212923 | -0.210004 | -0.135819 | 0.209065 |
| 3886 | 0.0217259 | -0.207116 | -0.137591 | 0.210281 |
| 3885 | 0.0212923 | -0.206672 | -0.140029 | 0.210723 |
| 3884 | 0.0212923 | -0.209782 | -0.139807 | 0.209618 |
| 3883 | 0.0208586 | -0.213559 | -0.13604 | 0.207959 |
| 3882 | 0.0212923 | -0.213559 | -0.132495 | 0.207959 |
| 3881 | 0.0221596 | -0.209782 | -0.13183 | 0.209507 |
| 3880 | 0.0225932 | -0.206005 | -0.134489 | 0.211276 |
| 3879 | 0.0221596 | -0.205561 | -0.13737 | 0.211497 |
| 3878 | 0.0212923 | -0.207783 | -0.138256 | 0.210391 |
| 3877 | 0.0199913 | -0.209116 | -0.137148 | 0.209507 |
| 3876 | 0.0195577 | -0.208005 | -0.136262 | 0.209728 |
| 3875 | 0.0199913 | -0.206894 | -0.137591 | 0.21017 |
| 3874 | 0.0208586 | -0.208005 | -0.139807 | 0.209728 |
| 3873 | 0.0212923 | -0.212004 | -0.139807 | 0.20818 |
| 3872 | 0.0212923 | -0.215559 | -0.13604 | 0.206522 |
| 3871 | 0.0208586 | -0.215559 | -0.132052 | 0.206633 |
| 3870 | 0.020425 | -0.212226 | -0.132052 | 0.208291 |
| 3869 | 0.0199913 | -0.211115 | -0.134268 | 0.209286 |
| 3868 | 0.0195577 | -0.214226 | -0.134711 | 0.208291 |
| 3867 | 0.0199913 | -0.216892 | -0.132273 | 0.206854 |
| 3866 | 0.020425 | -0.215337 | -0.130501 | 0.206964 |
| 3865 | 0.0208586 | -0.21156 | -0.132052 | 0.20818 |
| 3864 | 0.0212923 | -0.208894 | -0.134268 | 0.209286 |
| 3863 | 0.0212923 | -0.208005 | -0.134711 | 0.209618 |
| 3862 | 0.0212923 | -0.207783 | -0.133825 | 0.209618 |
| 3861 | 0.0212923 | -0.207338 | -0.133825 | 0.209839 |
| 3860 | 0.0208586 | -0.206894 | -0.135154 | 0.21017 |
| 3859 | 0.0208586 | -0.208005 | -0.136705 | 0.209728 |
| 3858 | 0.020425 | -0.211337 | -0.137591 | 0.208402 |
| 3857 | 0.020425 | -0.217558 | -0.137148 | 0.205638 |
| 3856 | 0.020425 | -0.225112 | -0.13604 | 0.202321 |
| 3855 | 0.0208586 | -0.23 | -0.135819 | 0.2 |
| 3854 | 0.0212923 | -0.227112 | -0.136705 | 0.201105 |
| 3853 | 0.0208586 | -0.220002 | -0.136705 | 0.204201 |
| 3852 | 0.020425 | -0.218669 | -0.134932 | 0.204975 |
| 3851 | 0.0195577 | -0.221113 | -0.132052 | 0.203869 |
| 3850 | 0.019124 | -0.218003 | -0.129836 | 0.204864 |
| 3849 | 0.0186904 | -0.210004 | -0.130501 | 0.208402 |
| 3848 | 0.0182567 | -0.203561 | -0.133381 | 0.211386 |
| 3847 | 0.0182567 | -0.201784 | -0.136262 | 0.212492 |
| 3846 | 0.0182567 | -0.202895 | -0.138035 | 0.212271 |
| 3845 | 0.0186904 | -0.205117 | -0.138699 | 0.211607 |
| 3844 | 0.0186904 | -0.207561 | -0.139142 | 0.210502 |
| 3843 | 0.0186904 | -0.210671 | -0.139364 | 0.209065 |
| 3842 | 0.0186904 | -0.214226 | -0.138478 | 0.207185 |
| 3841 | 0.0186904 | -0.217558 | -0.136483 | 0.205306 |
| 3840 | 0.019124 | -0.218669 | -0.133825 | 0.204311 |
| 3839 | 0.0195577 | -0.217336 | -0.132052 | 0.204643 |
| 3838 | 0.019124 | -0.21467 | -0.131387 | 0.205638 |
| 3837 | 0.0186904 | -0.213115 | -0.131387 | 0.206301 |
| 3836 | 0.0178231 | -0.213337 | -0.131166 | 0.206301 |
| 3835 | 0.0178231 | -0.213559 | -0.130501 | 0.206412 |
| 3834 | 0.0182567 | -0.212226 | -0.129836 | 0.207296 |
| 3833 | 0.0186904 | -0.209338 | -0.130058 | 0.208733 |
| 3832 | 0.0186904 | -0.206228 | -0.131387 | 0.21017 |
| 3831 | 0.0178231 | -0.20445 | -0.133381 | 0.210944 |
| 3830 | 0.0169558 | -0.20445 | -0.134711 | 0.211055 |
| 3829 | 0.0160885 | -0.204672 | -0.134711 | 0.210944 |
| 3828 | 0.0160885 | -0.204006 | -0.134268 | 0.211165 |
| 3827 | 0.0156548 | -0.203117 | -0.134932 | 0.211607 |
| 3826 | 0.0156548 | -0.204228 | -0.136927 | 0.211276 |
| 3825 | 0.0152212 | -0.208227 | -0.137813 | 0.209618 |
| 3824 | 0.0147875 | -0.213559 | -0.135819 | 0.207185 |
| 3823 | 0.0143539 | -0.216447 | -0.132717 | 0.205527 |
| 3822 | 0.0143539 | -0.215114 | -0.131609 | 0.205859 |
| 3821 | 0.0143539 | -0.21156 | -0.132717 | 0.207296 |
| 3820 | 0.0143539 | -0.211782 | -0.133603 | 0.207185 |
| 3819 | 0.0147875 | -0.215559 | -0.131166 | 0.205306 |
| 3818 | 0.0156548 | -0.21667 | -0.126955 | 0.204311 |
| 3817 | 0.0165221 | -0.211782 | -0.125626 | 0.206191 |
| 3816 | 0.0173894 | -0.205117 | -0.129393 | 0.209396 |
| 3815 | 0.0178231 | -0.202006 | -0.134268 | 0.211386 |
| 3814 | 0.0178231 | -0.202895 | -0.135819 | 0.211386 |
| 3813 | 0.0182567 | -0.203339 | -0.134711 | 0.211497 |
| 3812 | 0.0186904 | -0.201118 | -0.134268 | 0.212713 |
| 3811 | 0.0186904 | -0.19934 | -0.13604 | 0.213818 |
| 3810 | 0.0182567 | -0.200895 | -0.13737 | 0.213597 |
| 3809 | 0.0178231 | -0.203784 | -0.136927 | 0.212492 |
| 3808 | 0.0178231 | -0.204672 | -0.136262 | 0.211828 |
| 3807 | 0.0182567 | -0.204006 | -0.13737 | 0.211828 |
| 3806 | 0.0186904 | -0.205561 | -0.138921 | 0.210834 |
| 3805 | 0.0186904 | -0.210671 | -0.137813 | 0.208512 |
| 3804 | 0.0178231 | -0.215114 | -0.133603 | 0.206301 |
| 3803 | 0.0173894 | -0.214448 | -0.129836 | 0.206191 |
| 3802 | 0.0169558 | -0.20956 | -0.130501 | 0.207959 |
| 3801 | 0.0165221 | -0.205339 | -0.133603 | 0.209728 |

**Fig. S3.** FTIR spectra of crosslinked scaffolds between 1250-1150 cm^-1^. A) Gel scaffolds. B) Gel/PCL scaffolds; the doted rectangular regions highlight the peak at 1200 cm^-1^ (A) and 1190 cm^-1^ (B) due to presence of sulfate groups in the GAG-mimetic scaffolds which is not observed in Gel, Gel/PCL and PCL scaffolds.

**A**

**B**

**Fig. S4.** A) % swelling at each time point normalized to % swelling at initial 10-minute immersion; Scatter plot of individual data points where the means are connected between time points. The means are fold change with respect to 10 minutes of immersion; n=4 per group; * Significantly different for Gel/PCL/pCelS and Gel/PCL/fCelS (p<0.05) in comparison to Gel and Gel/PCL alone; ** Significantly higher at day 21 (504 hours) for Gel/fCelS as compared to all other scaffolds p<0.05). B) Representative SEM images of all scaffolds at 8 weeks (1,344 hours) of immersion; Mag.: 2000X; Scale bar: 1µm.

**Fig. S5.** Protein loss profile for all scaffolds over 8 weeks (56 days); n=4 per group; * Significantly less protein loss for Gel/PCL/fCelS group at days 4, 7, 14, 21, and 28 as compared to all other scaffolds (p<0.05); ** Significantly more protein loss for all Gel scaffolds in comparison to their corresponding Gel/PCL scaffolds at all time points (p<0.05). Scatter plot of individual data points where the means are connected between each time point.

**A**

**B**

**C**

**D**

**Fig. S6.** Neurotrophic binding and release. A) NT-3 and B) BDNF binding after 24 hours; n = 5 per group; Groups with different letters are statistically significant (p<0.05). Column scatter plot where the bar and error bars represent Mean ± S.D.. Cumulative release profile of C) NT-3 and D) BDNF; ** fCelS containing groups are significantly lower than all other groups at each time point (p<0.05); n=5 per group. Scatter plot of individual data points where the means are connected between each time point.

**A**

Fibers

Merged

NF

**B**

**C**

**Fig. S7.** A) Representative merged confocal images of DRG explants cultured on all scaffolds at 3 days; Scale bar: 250 µm. Green: Neurofilament. B) A representative confocal image on Gel/fCelS scaffolds at Mag. 60X; Scale bar: 50 µm; Green: Neurofilament; Blue: Dapi, Fibers autofluoresce in blue. C) Axon length on all scaffolds; n=4 per group where each data point represents the average of 10 axons per sample; Groups with different letters are statistically significant (p<0.05). Column scatter plot where the bar and error bars represent the Mean ± S.D..

**Fig. S8.** Representative confocal images of eGFP-SCs on all scaffolds at day 14. Mag.: 20X; Scale bar: 100 µm.
